# Changes in growth, lanthanide binding, and gene expression in *Pseudomonas alloputida* KT2440 in response to light and heavy lanthanides

**DOI:** 10.1128/msphere.00685-24

**Published:** 2024-09-18

**Authors:** Linda Gorniak, Sarah Luise Bucka, Bayan Nasr, Jialan Cao, Steffen Hellmann, Thorsten Schäfer, Martin Westermann, Julia Bechwar, Carl-Eric Wegner

**Affiliations:** 1 Institute of Biodiversity, Aquatic Geomicrobiology, Friedrich Schiller University Jena, Jena, Germany; 2 Department of Physical Chemistry and Microreaction Technology, Institute for Chemistry and Biotechnique, Technische Universität Ilmenau, Ilmenau, Germany; 3 Institute of Geosciences, Applied Geology, Friedrich Schiller University Jena, Jena, Germany; 4 International Max Planck Research School for Global Biogeochemical Cycles, Department of Biogeochemical Processes, Max Planck Institute for Biogeochemistry, Jena, Germany; 5 Electron Microscopy Center, Jena University Hospital, Jena, Germany; 6 Bioinorganic Chemistry, Heinrich Heine University Düsseldorf, Düsseldorf, Germany; The University of Iowa, Iowa City, Iowa, USA

**Keywords:** lanthanides, lanthanome, RNAseq, microfluidics, single-cell ICP-MS

## Abstract

*Pseudomonas alloputida* KT2440 is a ubiquitous, soil-dwelling bacterium that metabolizes recalcitrant and volatile carbon sources. The latter is utilized by two redundant, Ca- and lanthanide (Ln)-dependent, pyrroloquinoline quinone-dependent alcohol dehydrogenases (PQQ ADH), PedE and PedH, whose expression is regulated by Ln availability. *P. alloputida* KT2440 is the best-studied non-methylotroph in the context of Ln-utilization. Combined with microfluidic cultivation and single-cell elemental analysis, we studied the impact of light and heavy Ln on transcriptome-wide gene expression when growing *P. alloputida* KT2440 with 2-phenylethanol as the carbon and energy source. Light Ln (La, Ce, and Nd) and a mixture of light and heavy Ln (La, Ce, Nd, Dy, Ho, Er, and Yb) had a positive effect on growth, whereas supplementation with heavy Ln (Dy, Ho, Er, and Yb) exerted fitness costs. These were likely a consequence of mismetallation and non-utilizable Ln interfering with Ln sensing and signaling. The measured amounts of cell-associated Ln varied between elements. Gene expression analysis suggested that the Ln sensing and signaling machinery, the two-component system PedS2R2 and PedH, responds differently to (non-)utilizable Ln. We expanded our understanding of the lanthanide (Ln) switch in *P. alloputida* KT2440, demonstrating that it adjusts the levels of *pedE* and *pedH* transcripts based on the availability of Ln. We propose that the usability of Ln influences the bacterium’s response to different Ln elements.

**IMPORTANCE:**

The Ln switch, the inverse regulation of Ca- and Ln-dependent PQQ ADH in response to Ln availability in organisms featuring both, is central to our understanding of Ln utilization. Although the preference of bacteria for light Ln is well known, the effect of different Ln, light and heavy, on growth and gene expression has rarely been studied. We provide evidence for a fine-tuning mechanism of Ca- and Ln-dependent PQQ ADH in *P. alloputida* KT2440 on the transcriptome level. The response to (non-)utilizable Ln differs depending on the element. Ln commonly co-occur in nature. Our findings underline that Ln-utilizing microbes must be able to discriminate between Ln to use them effectively. Considering the prevalence of Ln-dependent proteins in many microbial taxa, more work addressing Ln sensing and signaling is needed. Ln availability likely necessitates different adaptations regarding Ln utilization.

## INTRODUCTION

Together with the chemically similar elements scandium (Sc) and yttrium (Y), the lanthanides (elements 57–71 [La–Lu], Ln) are commonly known as “rare-earth elements” or more precisely, “rare-earth metals” (REM). Based on their electron configuration, which governs interactions with other elements, Ln are grouped into light (La–Eu, Sc; LREM) and heavy (Gd–Yb, Y; HREM) REM (1, 2). Ln are key resources for numerous high-tech applications and are in increasing demand for the ongoing green energy transition (3). The lack of sustainable mining and recycling methods puts a spotlight on understanding Ln-dependent metabolism as a foundation for biology-inspired Ln recovery strategies.

Our knowledge about Ln-dependent metabolism is primarily derived from methylotrophic bacteria, which utilize C_1_ compounds as carbon and energy sources (4, 5). The first discovered group of Ln-dependent enzymes, Xox-type methanol dehydrogenases (MDHs), catalyzes the oxidation of methanol to formaldehyde. The role of Xox-type MDH was long unclear, and calcium-dependent Mxa-type MDHs were considered the *de facto* key enzymes for methanol oxidation in methylotrophs (6, 7). Xox- and Mxa-type MDHs belong to the diverse group of pyrroloquinoline quinone (PQQ)-dependent alcohol dehydrogenases (ADH) (8). Genes encoding Xox-type MDHs were detected in many environments and also in non-canonical methylotrophs (9, 10), suggesting that methylovory (the supplemental use of C_1_ compounds as energy sources) is more common than anticipated (11). The PQQ ADH family includes over a dozen subclades (8). Most are assumed to rely on Ln. In bacteria possessing pairs of Ca- and Ln-dependent ADH, the expression of the corresponding genes is inversely regulated by the “Ln switch,” which is rooted in two-component systems and auxiliary proteins (12–17).

Microbes prefer LREM (5). Mechanisms underlying Ln mobilization and uptake likely differ depending on local Ln bioavailability. Work in the model methylotroph *Methylorubrum extorquens* identified two gene clusters, the *lut*-cluster (lanthanide utilization and transport) and the *mll-*/*mlu-*cluster (methylolanthanin uptake), which encode uptake machinery centered around a TonB-dependent receptor and an ABC transporter (18), and biosynthetic machinery for a Ln-binding metal chelator (19). Ln commonly co-occur in the environment. It is not clear how Ln-utilizing microbes distinguish between them. Using Beijerinckiaceae bacterium RH AL1, we could show that Ln are selectively taken up and that supplementation with different Ln elements had only minor effects on growth but substantial effects on gene expression (20).

*Pseudomonas alloputida* KT2440 is the best-studied, non-methylotroph in the context of microbial Ln utilization (15, 21–23). Strain KT2440 is characterized by a broad metabolic versatility, including the potential to use volatile organic compounds (VOC) that carry alcohols or aldehydes as functional groups (24–26). The utilization of VOC is facilitated by two inversely regulated, broad-range PQQ ADH PedH (Ln-dependent) and PedE (Ca-dependent). In *P. alloputida* KT2440, the Ln switch is rooted in the interplay between the Ca-dependent PQQ ADH PedE and the Ln-dependent PQQ ADH PedH (21). PedH also functions as a sensory module and is, together with the PedS2R2 (PP_2671, PP_2672) two-component system (15), integral for Ln signaling. It was previously postulated that PedS2 phosphorylates its LuxR-type response regulator PedR2 in the absence of La, which in turn activates *pedE* and limits *pedH* expression (15). If La is available, PedS2 kinase activity is reduced, *pedE* expression is decreased, and *pedH* transcription is activated (15).

In this study, we investigated the effect of Ln supplementation on growth, cell-associated Ln, and gene expression. Our findings indicate that the Ln switch fine-tunes the *pedE* and *pedH* transcript pools in *P. alloputida* KT2440. Differences in the response to (non-)utilizable elements suggest that *P. alloputida* KT2440 can distinguish Ln elements to effectively use them for its metabolism.

## RESULTS

### Effect of La concentration and Ln elements on growth

**Fig 1:**
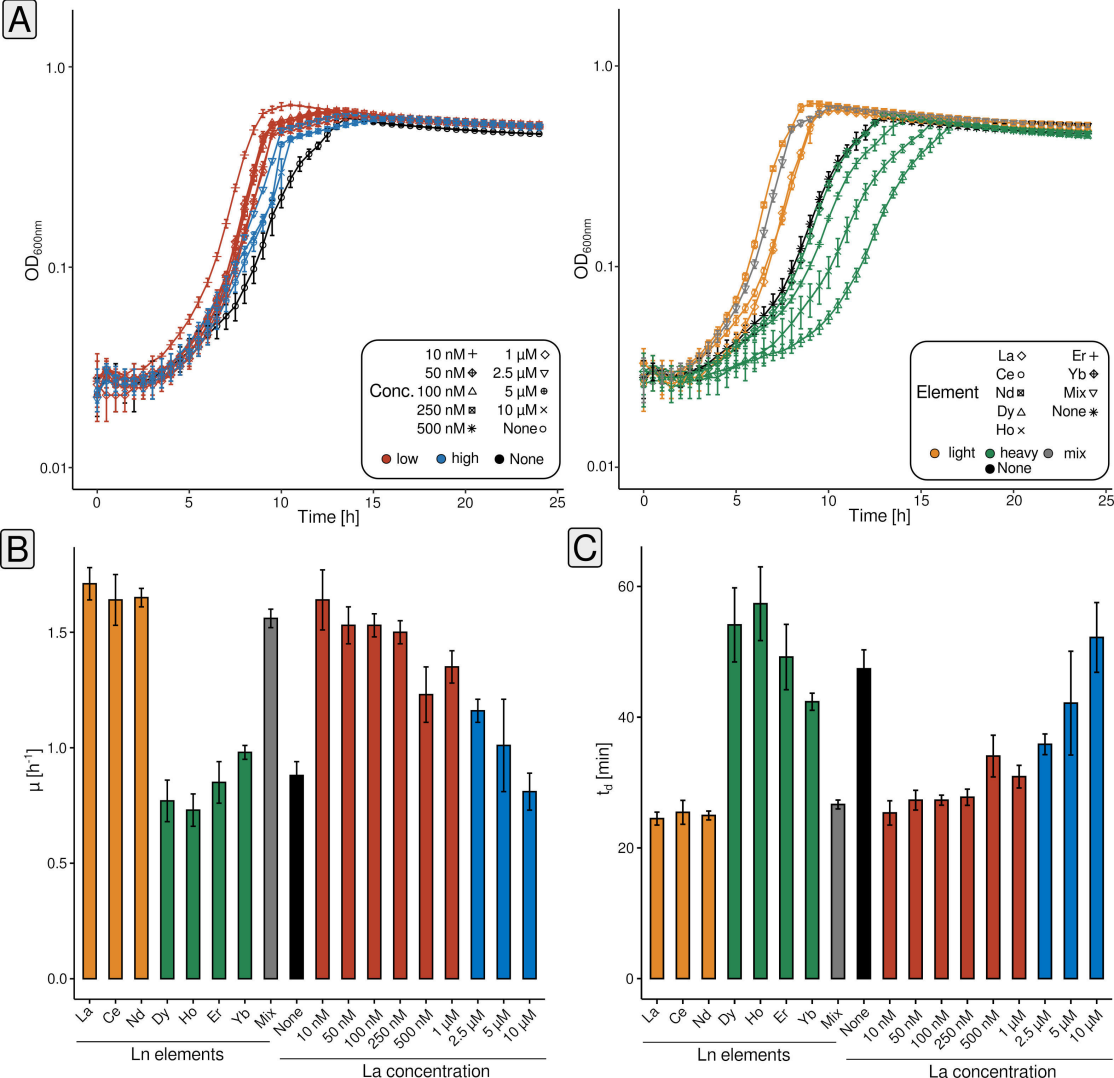
Assessing the growth of *P. alloputida* KT2440 with different concentrations of La (10 nM - 10 µM) and different Ln elements (50 nM). Cultivations have been carried out in 96-well plates (*n* = 4 biological replicates) in 300 µL MP medium, and growth was tracked through spectrophotometry (OD_600_) (**A**). The symbols and color codes refer to different La concentrations, and different Ln elements, respectively (red = low concentrations, 10 nM–1 µM; blue = high concentrations, 2.5–10 µM; orange = light Ln, La–Nd; green = heavy Ln, Dy–Yb; grey = Ln mix [Ce, Nd, Dy, Ho, Er, and Yb]; black = no added Ln). Growth rates and doubling times were calculated based on averaged data using growthcurver (v0.3.1) (27) (**B**). Obtained growth curve data were fitted based on the standard form of the logistic equation in ecology and evolution, which is rooted in growth rate, initial population size, and carrying capacity (28, 29).

We first grew *P. alloputida* KT2440 with different La concentrations (10, 50, 100, 250, and 500 nM, 1, 2.5, 5, and 10 µM; and without added Ln) (Fig. 1A, left panel). The lag phase was shortest with 10 and 50 nM La. A maximum growth rate (*r*) of 1.64 ± 0.13 h^−1^ for the corresponding cultures was estimated, almost twice as high as for those without added Ln (Table S1). The shortest estimated doubling time (*t*_*d*_) has been 0.42 ± 0.03 h in the case of the 10 nM cultures (Fig. 1B). Except for 10 µM, all tested concentrations had a positive effect on *r* and *t*_*d*_ (Table S1).

We compared the effect of 50 nM of different Ln elements on growth (La, Ce, Nd, Dy, Ho, Er, Yb, and Mix [equimolar mix of Ce, Nd, Dy, Ho, Er, and Yb; 50 nM total Ln]) (Fig. 1A, right panel). The lag phase was the shortest for Nd cultures. The lighter Ln (La, Ce, and Nd) and the Ln mix had a positive effect on growth when compared with cultures without added Ln (Fig. 1B and C). The estimated growth rates were between 1.71 ± 0.07 (La) and 1.56 ± 0.04 h^−1^ (Ln mix), with doubling times around 0.4 h (Table S1). The growth rate was 0.88 ± 0.06 h^−1^ without added Ln, and the doubling time was 0.79 ± 0.05 h. Heavier Ln impaired growth. Lag phases and doubling times were longer, and growth rates were decreased. Yb cultures almost behaved like those without added Ln.

### Microfluidic one- and two-dimensional screenings with light and heavy Ln

**Fig 2:**
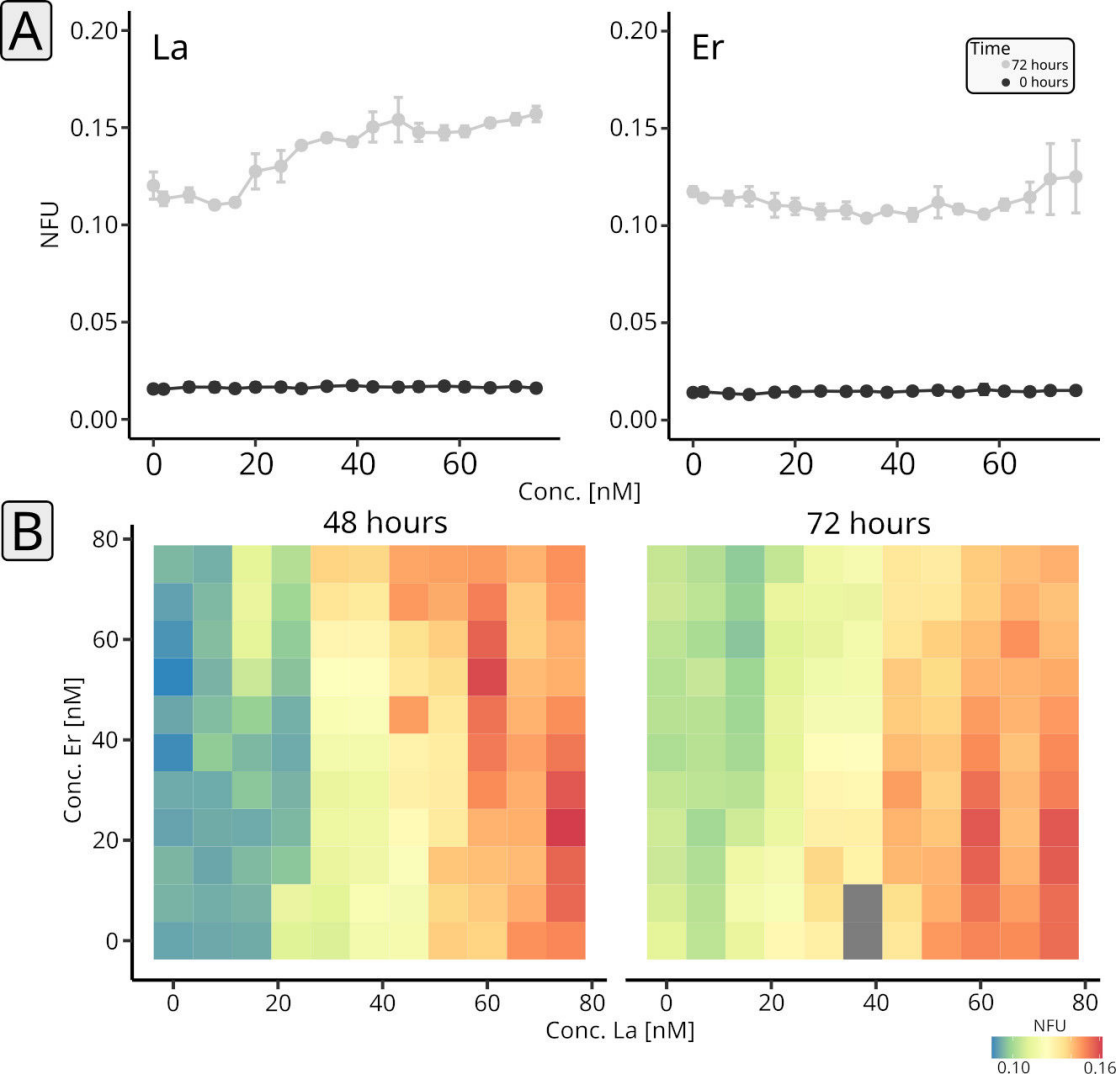
One- and two-dimensional microfluidic cultivation of *P. alloputida* KT2440 with different Ln elements. One-dimensional screenings have been carried out for La and Er ramping from 0 to 75 nM, over 18 steps (*n* = 3–5 microfluidic segments per concentration, technical replicates). Per Ln element, two or three tube coils were incubated (*n* = 2–3, biological replicates). Incubations were done for 72 h. Plotted are normalized autofluorescence units (NFU) and standard deviations at the beginning (black) and end of the incubations (gray) (**A**). A two-dimensional screening has been done for La vs Er (**B**). We tested 121 combinations of concentrations (0–75 nM) against each other and incubated the corresponding microfluidic segments for three days. Per combination, between three and five microfluidic segments were generated (technical replicates). Plotted are NFU values and standard deviations after two and three days. For the measurements, complete sequences were moved through the sensors. Especially due to repeated measurements, it can happen that some droplets merge (see supplemental material). For the evaluation, only droplets that have not merged have been considered. Grayed-out tiles refer to droplets that suffered from droplet fusion.

We established microfluidic cultivation (Fig. S1) using segmented flow, based on a previously validated setup (30) (Fig. S2), for *P. alloputida* KT2440 in 500 nL droplets in PP9 (perfluoromethyldecalin) carrier medium, stored, and incubated in polytetrafluoroethylene (PTFE) tubing. The composition concerning added Ln was manipulated, yielding either a series of droplets characterized by increasing Ln concentration (0–75 nM) (one-dimensional screenings, Fig. 2A), or a series of droplets featuring different concentration ratios of two different Ln elements (0–75 nM, two-dimensional screenings, Fig. 2B).

*P. alloputida* KT2440 reached the stationary phase in the droplets after 72 h (data not shown). Growth was monitored by measuring the autofluorescence (405 nm excitation and 425 nm emission). In the case of La, the maximum attained autofluorescence after 3 days of incubation increased with La concentration, but only marginally for concentrations above 50 nM (Fig. 2A, left panel). Er had no impact on attained autofluorescence values, but higher concentrations were reflected in a higher observed variability (Fig. 2A, right panel).

We carried out a two-dimensional screening (La vs. Er) to study how the interplay between light and heavy Ln affects growth (Fig. 2B; Table S2). After 48 h, the highest autofluorescence values were observed for the combinations of 60 nM La and 52.5 nM Er, and 75 nM La and 30 nM Er, respectively (Fig. 2B). Overall, autofluorescence values tended to be higher with increasing La concentration. We did not see any interaction between the two effectors, La and Er. Additionally, added Er did not, or only marginally, impact growth.

### Analysis of cell-bound Ln using single-cell elemental analysis

The observed effects of different Ln elements on growth prompted us to probe *P. alloputida* KT2440 for Ln accumulation and uptake. Using transmission electron microscopy (TEM) and energy-dispersive X-ray spectroscopy (EDX), we could neither identify extra- nor intracellular Ln accumulations when 50 nM or 1 µM Ln was added. Inspecting and measuring cell aggregates instead of individual cells did not yield any Ln-positive signals (Fig. S3). To assess cell-associated Ln, we made use of single-cell inductively coupled mass spectrometry (scICP-MS). We measured La, Nd, and Er starting from fixed biomass from incubations supplemented with an excess of Ln (1 µM of the respective element), and measured Ce, Nd, and Er in the case of the Ln mix.

**Fig 3:**
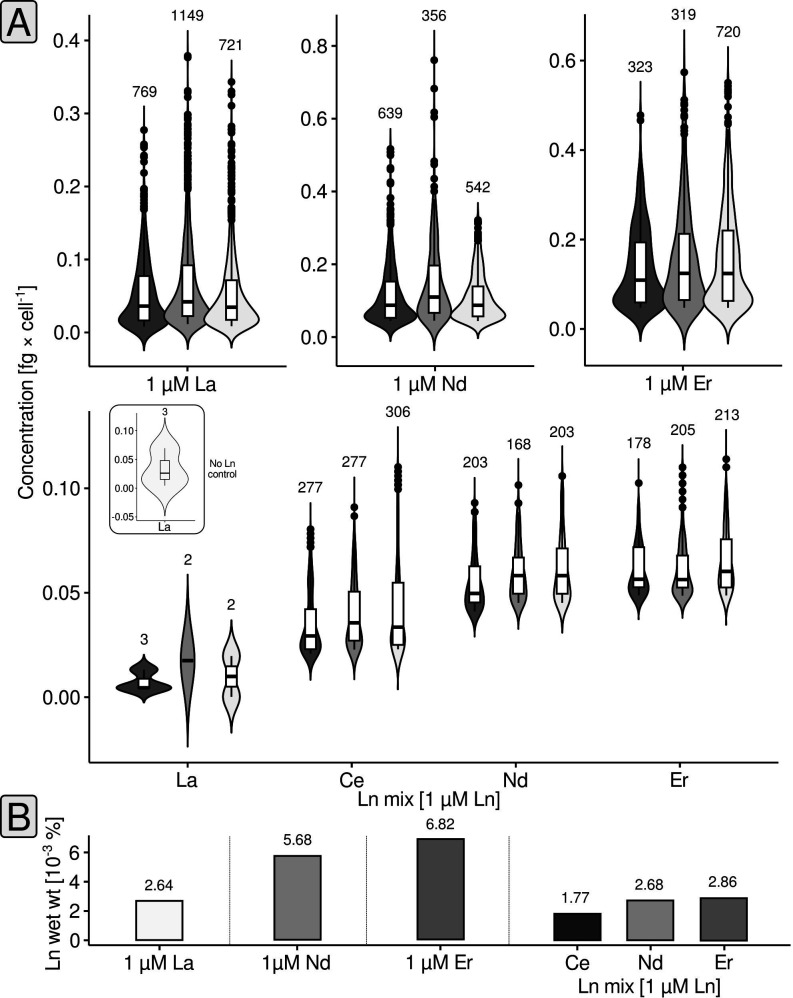
Single-cell elemental analysis. The Ln concentration per cell is shown through combined violin and box plots (**A**). The different shades of gray indicate biological replicates (*n* = 3). The box plots indicate lower and upper quartiles, as well as median values. Black circles represent cells with concentrations above (or below) the whisker limits. The numbers above the violin plots indicate the number of considered events (= cells). The small subpanel shows the results of the control sample without any added Ln. Sporadically detected La events in the case of the control, and Ln mix samples represent background noise of the measurement. The %Ln of the wet weight (**B**) was calculated as outlined in Materials and Methods.

For each sample, several hundred events (= cells) have been analyzed (Fig. 3A). Cells grown with 1 µM La featured on average 0.058 ± 0.055 fg La cell^−1^. The amount of cell-associated Ln for cells grown with either Nd or Er was higher and around 0.125 ± 0.086 fg Nd cell^−1^ and 0.152 ± 0.106 fg Er cell^−1^. Assuming an average wet weight per cell of 2.2 pg (see Materials and Methods), between 2.64 × 10^−3^ (La) and 6.82 × 10^−3^% (Er) of the wet weight were made up by cell-associated Ln (Fig. 3B). For the Ln mix samples, we observed that Ln made up a slightly higher proportion of the wet weight (7.31 × 10^−3^%), and we could see that the amount of cell-associated Ln differed between Ln elements. We detected on average 0.039 ± 0.018 fg Ce cell^−1^ (1.77 × 10^−3^% cellular wet weight), 0.059 ± 0.037 fg Nd cell^−1^ (2.68 × 10^−3^%), and 0.063 ± 0.014 fg Er cell^−1^ (2.86 × 10^−3^%).

### Overall effects of ln elements on gene expression

**Fig 4:**
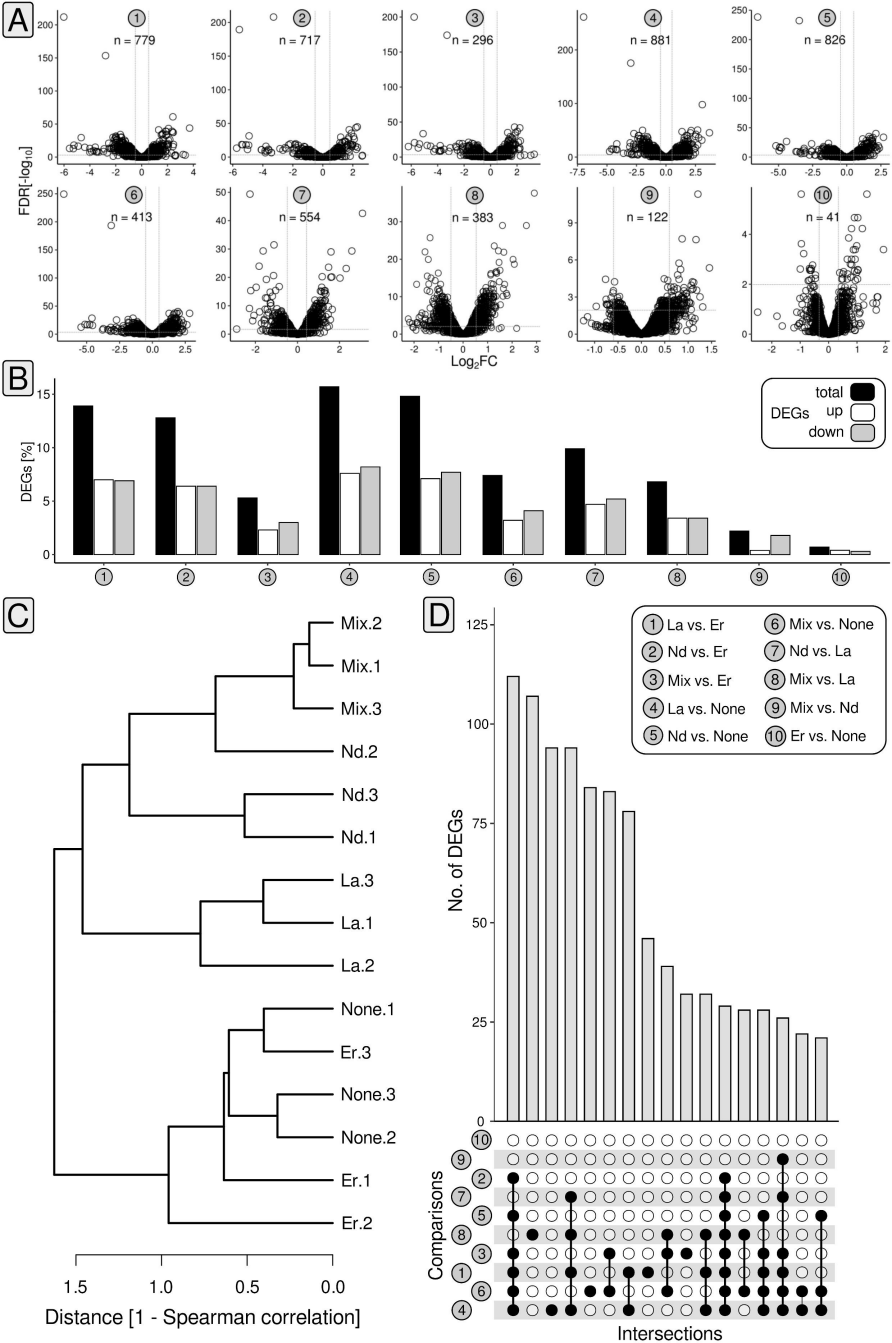
Statistics and summary of gene expression analysis. Volcano plots summarize the number of differentially expressed genes (DEGs) and the breadth of observed fold changes for the different comparisons (1–10). We defined differential gene expression based on changes in expression (> |0.58| log_2_FC [fold change]), relative gene expression (> 4 log_2_[counts per million [CPM]), and FDR-corrected *P*-values (<0.5). Gray dashed lines indicate the log_2_FC threshold of |0.58| (**A**). The proportion of DEGs was summarized as bar charts. The color code refers to the proportion of differentially expressed (black), upregulated (white), and downregulated genes (gray) (**B**). Based on the counts per million (CPM) matrix, inter-sample relationships have been determined by calculating Spearman distances (**C**). Shared genes between the different comparisons were highlighted using UpSet plots (**D**).

Starting from mid-exponential phase biomass samples (*t*_1_ + *t*_2_, Fig. S4) originating from cultures grown with 50 nM of different Ln elements (La, Nd, Er, Mix [equimolar mix of Ce, Nd, Dy, Ho, Er, Yb; 50 nM total Ln]) or without Ln, we performed RNAseq-based (Table S3) differential gene expression analysis (DGEA). We considered genes with changes in the expression above |0.58| log_2_fold change (FC; a log_2_FC > |0.58| is equivalent to gene expression changes > 50%), expression values higher than four log_2_CPM (counts per million), and false-discovery rate (FDR)-adjusted *P*-values < 0.05 as differentially expressed (Fig. 4A; Tables S4 to S13). We determined differentially expressed genes (DEGs) for a total of 10 different comparisons: (1) La vs. Er, (2) Nd vs. Er, (3) mix vs. Er, (4) La vs. none, (5) Nd vs. none, (6) mix vs. none, (7) Nd vs. La, (8) mix vs. La, (9) mix vs. Nd, and (10) Er vs. none (Fig. 4A).

The number of DEGs ranged between 41 (Er vs. none) and 881 (La vs. none), which translates into 0.7% and 15.7% of the *P. alloputida* KT2440 protein-encoding genes (Fig. 4B). Replicate data sets from the incubations with Er and without added Ln clustered together. The same was true for the La, Nd, and Ln mix replicates (Fig. 4C).

We determined the overlap between individual comparisons (Fig. 4D) and noted the biggest overlap of 112 DEGs when looking at comparisons 1–6. Subsequently, 94 shared DEGs were observed in the case of comparisons 1, 4, 7, and 8, and 28 genes were differentially expressed in all comparisons except 9 and 10. There were no DGEs that were shared by all comparisons.

### Differential gene expression and cluster analysis

**Fig 5:**
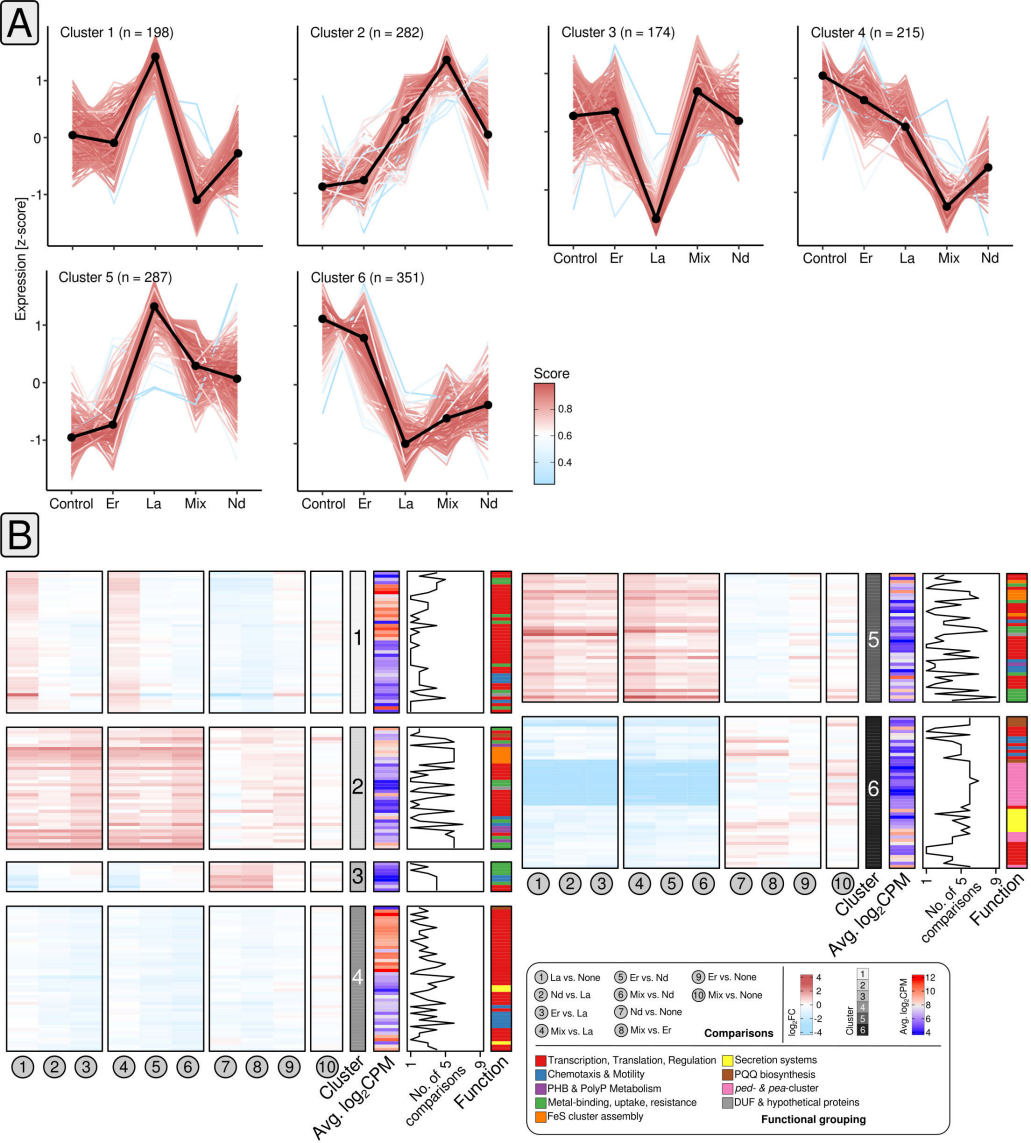
Cluster and differential gene expression analysis. *K*-means clustering (31) was used to identify clusters of genes with matching gene expression patterns (**A**). The number of clusters was estimated *a priori* through Spearman distances, the Calinski–Harabasz index (32), the sum of squared errors, the average silhouette width (33), and the gap statistic (34) (Fig. S5). We ultimately settled on *K* = 6. The color code indicates the Pearson correlation between each gene of the respective cluster and the cluster centroid (black). Changes in gene expression of cluster genes for each comparison (1–10) are shown for selected subsets of genes (**B**). The heatmap color codes refer to log_2_FC and average log_2_CPM values. The functional annotation of the genes is color-coded as well. The line plot indicates how often the genes were considered as differentially expressed based on the different comparisons.

We used K-means clustering (31) for grouping differentially expressed genes based on co-expression. We evaluated different algorithms (Fig. S5) for estimating *K*, and ultimately settled on *K* = 6. Inspecting gene expression by determined z-scores, revealed pairs of clusters characterized by inversed gene expression patterns (clusters 1 and 3, 2 and 4, 5 and 6) (Fig. 5A).

We noted that cluster 1 genes not only responded to La supplementation by increased gene expression compared with Er and cultures without Ln, but also reduced gene expression when comparing La to Nd and the Ln mix. Cluster 1 included ribosomal protein (PP_0445–46) and transcriptional regulator (PP_4476) genes (Fig. 5B). We also noticed genes linked to spermidine/putrescine transport (PP_0412–13) and a gene coding for a DUF2790 domain-containing protein. The latter was strongly downregulated when comparing La supplemented cultures with those grown with Er or without Ln (log_2_FC −2.89,–2.62).

When looking at cluster 2, gene expression was elevated in all comparisons between cultures grown with La, Nd, or the Ln mix; and cultures supplemented with Er or no Ln. A weaker response was visible when Ln mix cultures were checked against, La, Nd, or Er. Cluster 2 comprised transcriptional regulator genes (PP_1683, PP_3756), genes encoding (heavy) metal sensor and transport proteins (PP_5139, PP_5383–84), a phasin gene (PP_5008), but also genes involved in Fe–S cluster assembly (PP_0843–46) (Fig. 5B).

Cluster 3 included two gene copies encoding the chemotaxis-related adaptor protein CheW (PP_1489, PP_1491). It featured reduced expression in the two comparisons between La and Er and between La and the control cultures, whereas expression was higher when contrasting Nd, and the Ln mix, against La. The genes belonging to cluster 4 included multiple TetR/Acr transcriptional regulator genes (PP_1961, PP_1968), as well as various ribosomal protein genes. We noted lower gene expression when comparing Nd and Ln mix cultures with Er and cultures without added Ln.

The addition of light/mixed Ln caused elevated gene expression of cluster 5 genes compared with the control set-up and cultures grown with Er. Genes for FeS-cluster assembly proteins were sensitive to Ln supplementation (log_2_FC −1.78 to 1.76), whereas the most responsive cluster 5 genes encoded metal-sensing, metal-binding proteins, and another DUF2790 domain-containing protein (PP_2969, PP_5732, PP_3494; log_2_FC −3.34 to 3.04). Cluster 6, covered in more detail in the next section, included lanthanome-related genes.

### Differential gene expression of lanthanome-associated genes

**Fig 6:**
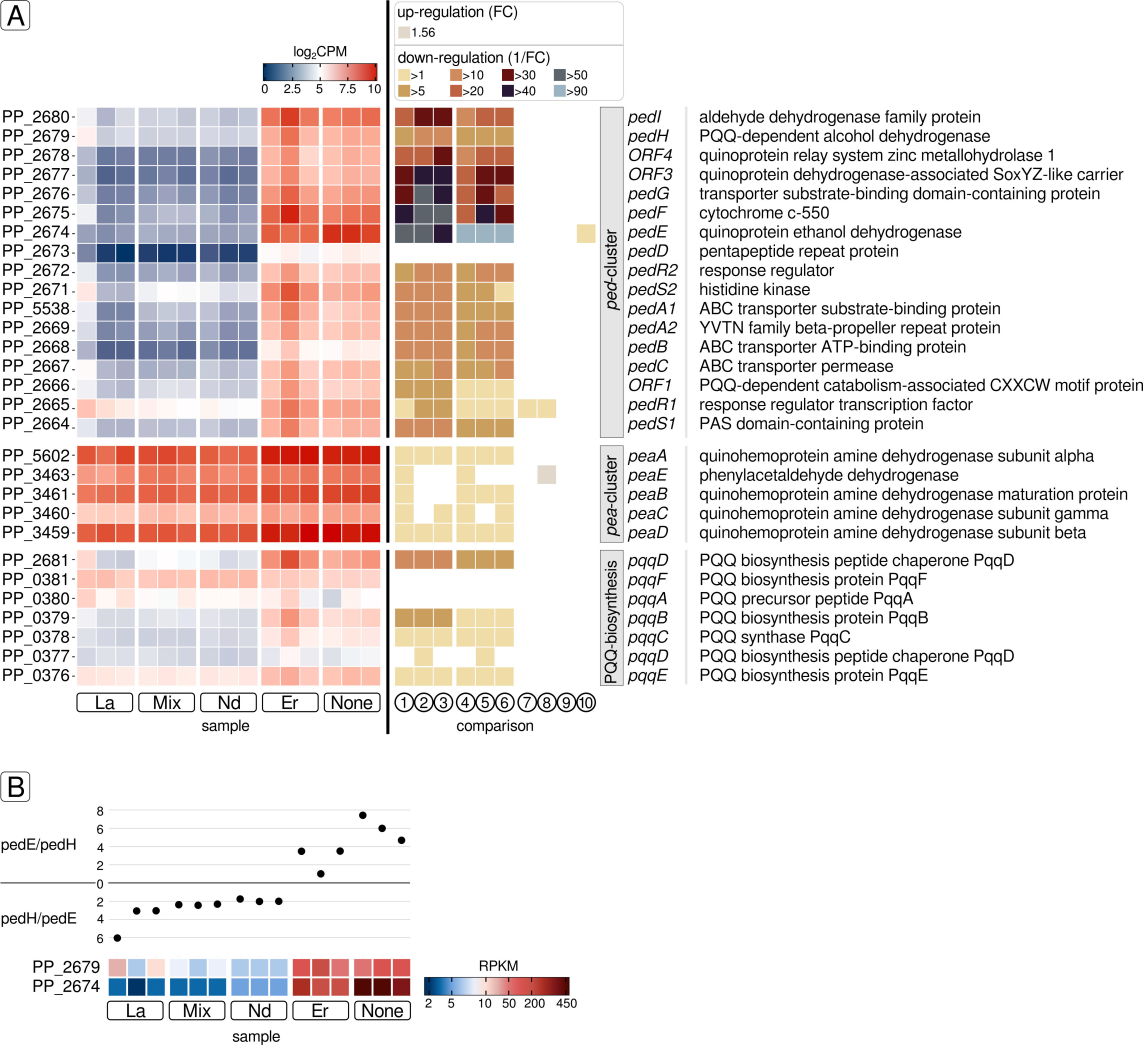
Gene expression analysis of lanthanome-related genes. We considered the *ped*-cluster, the *pea*-cluster, and genes involved in PQQ biosynthesis as part of the lanthanome. Gene expression is plotted based on log_2_CPM values (A, left panel). Changes in gene expression are shown for each comparison (1 to 10 as described in Fig. 5) based on the respective fold change (FC) or its reciprocal (1/FC) in case of downregulation (A, right panel). The latter value describes the factor by which the expression of the respective gene is reduced. The Ln element-dependent gene expression ratios of the PQQ ADH-encoding genes *pedE* (PP_2674) and *pedH* (PP_2679) were calculated based on RPKM values (**B**).

The lanthanome comprises all biomolecules, primarily proteins, directly involved in Ln utilization (35). In *P. alloputida* KT2440, Ln utilization is linked to the *ped*-cluster (PP_2664 – PP_2680), which is associated with 2-phenylethanol uptake and its conversion to phenylacetic acid (36). It includes *pedE*, *pedH*, as well as genes coding for the Ln-sensing two-component system PedS2/PedR2 (PP_2671/PP_2672), and an ABC transporter PedA1A2BC (PP_5538, PP_2669, PP_2668, PP_2667) facilitating cytoplasmic Ln uptake. Except for Er addition, the *ped*-cluster, including *pedE* and *pedH*, was downregulated upon Ln supplementation (Fig. 6A; Tables S4 to S13) (log_2_FC values between −1.29 and −6.98). Only *pedE* (PP_2674) was differentially expressed when comparing incubations with Er and without added Ln (log_2_FC −0.96). Depending on the added Ln element, *pedE* gene expression differed by more than two orders of magnitude (fold changes of up to 126). Its highest gene expression was detected in samples without Ln (reads per kilobase per million [RPKM] 410.28 ± 56.55), followed by Er (RPKM 210.45 ± 27.53). Significantly lower gene expression was observed in samples supplied with light Ln or the Ln mix. Gene expression ranged between 4.45 ± 0.29 (Nd) and 3.25 ± 0.57 (La) RPKM. The gene expression ratio of *pedE* and *pedH* differed depending on the Ln supply (Fig. 6B). In samples without Ln or with added Er, the *pedE:pedH* gene expression ratio was six and two, respectively. The *pedE:pedH* ratio shifted in favor of *pedH* in response to La, Nd, and the Ln mix. The expression of *pedH* was between 1.9-fold (Nd) and 4.2-fold (La) higher. The gene expression of *pedE* and *pedH* was additionally investigated via reverse transcription qPCR (RT-qPCR) from total RNA samples and making use of the 2^-ΔΔCt^ method (37). For this purpose, cultivation was repeated for selected Ln elements (La, Er, controls without Ln), and samples for total RNA extraction were taken from the mid-exponential growth phase (OD_600_ between 0.29 ± 0.06 [no Ln] and 0.31 ± 0.01 [Er]). Compared with samples without added Ln (controls), La samples showed a 2.4-fold higher expression of *pedH* (Fig. S6). Samples with Er or without Ln, featured comparable *pedH* expression. Er samples showed higher gene expression of *pedE* compared with controls without Ln.

Besides the *ped*-cluster, we also considered PQQ biosynthesis as part of the lanthanome. We noted a downregulation of PQQ biosynthesis-related genes (*pqqBCDE* [PP_0379, PP_0378, PP_0376, PP_2681]) when comparing La, Nd, and the Ln mix against Er and samples without Ln addition. Differential gene expression was not detected when light Ln samples were compared with each other or the Ln mix. There was no difference between the Er and no Ln samples. In addition, we looked at the *pea*-cluster (encoding genes of the 2-phenylethylamine pathway) since encoded proteins include quinoprotein dehydrogenases. So far, all known Ln-dependent enzymes are quinoprotein dehydrogenases, and we were wondering if genes belonging to the pea-cluster are responding to Ln supplementation. Genes associated with the *pea*-cluster (PP_3459–PP_3461, PP_5602) featured expression values between 6.21 ± 0.06 and 10.06 ± 0.09 log_2_CPM. Downregulation was observed when light Ln or the Ln mix was supplied (log_2_FC between −0.60 and −1.29).

## DISCUSSION

Our findings suggest that the Ln sensing and signaling machinery in *P. alloputida* KT2440, encoded by *pedE*, *pedH*, and *pedS2R2*, responds differently to utilizable and non-utilizable Ln. Utilizable Ln reduce Ln signaling-related gene expression. A smaller pool of *pedS2R2* transcripts presumably results in a reduction of PedS2 kinase activity, less phosphorylated PedR2, and ultimately reduced *pedE* expression. It is not clear if PedS2 binds heavier Ln, if heavier Ln reduce its activity, or if any other sensory elements are acting upstream of PedS2R2. Compared with cultures without added Ln, we did not observe an upregulation of *pedH* in response to Ln supplementation in RNAseq data sets. Except for Er, we noted the simultaneous downregulation of *pedE* and *pedH* in the presence of Ln, but also the reversion of their expression ratio. We also assessed changes in *pedE* and *pedH* expression by means of RT-qPCR, but had to use sample material from an independent incubation, due to a lack of original sample material. RT-qPCR revealed that *pedH* was upregulated in response to La, whereas its expression was stable, but not reduced, with added Er. The opposite was true for *pedE*. Expression was higher when Er was added and did not change significantly in the presence of La. Comparing RT-qPCR and RNAseq-derived data has inherent shortcomings. Different sets of incubations introduce biological variability. Both methods differ regarding sample processing. Factors, such as RNA quality, reverse transcription, and PCR efficiency add further uncertainty. Nonetheless, our data collectively show that the Ln switch is not acting as a simple on-off switch on the transcriptional level. Previously carried-out promoter activity assays (21) showed an increase of *pedH* and a decrease of *pedE* promoter activity in the presence of La.

Modulating the pool of PQQ ADH depending on Ln availability might be a mechanism to optimize substrate utilization. PedH features a higher *V*_max_ and specific activity compared with PedE. At the same time, PedH has a much higher affinity for Ln than PedE for Ca (21). The downregulation of *pedE*, while *pedH* expression was unaltered when *P. alloputida* KT2440 was supplemented with Er, supports the idea that non-utilizable Ln might interfere with PedSR2 activity and the modulation of the PQQ ADH pool. Enzymatic activity assays have shown that PedH activity is highest with LREM and absent with heavy Ln, such as Er or Yb (21). Ln-dependent PQQ ADH can accommodate LREM and HREM with only minor changes in overall protein structure. However, differences in ionic radii and coordination number affect the catalytic efficiency of PQQ ADH and are potential reasons for bacteria preferring the larger and more abundant LREM (38). Accidental mismetallation of PedH with non-utilizable Ln and the reduced transcript pool of the second PQQ ADH, PedE, might explain the impaired growth observed with Er.

Droplet-based microfluidics combined with segmented flow is ideally suited for high-throughput cultivation and has been used for cultivating and characterizing various microbes, for instance, soil microorganisms, such as *Rhodococcus* sp. and *Chromobacterium* (39), cyanobacteria (40), as well as plant stem cells (41). Our one-dimensional microfluidic screenings suggested higher fitness with increasing La concentration, which matched the observed growth efficiency when using conventional cultivation. Considering that PedH is not active with Er (21), and changes in gene expression, the observed increase in autofluorescence variability for higher Er concentrations did not reflect higher fitness. Instead, it might be an indicator of increased oxidative stress, indicating a reduced overall fitness. Cellular autofluorescence in bacteria is primarily linked to NADH (NADH is fluorescent, emission spectrum 420–550 nm; NAD is not fluorescent) and, to a lesser extent, flavins. More noisy autofluorescence values suggest an effect on the NADH pool, which is easily affected by oxidative stress (42, 43).

Conventional cultivation in bottles has shown that supplementation with Er has a slight negative effect on growth. The performed two-dimensional screening suggested that potential negative effects mediated by Er were masked and compensated by the added La when strain KT2440 was exposed to both. This was in line with the positive effect seen for bottles supplemented with the Ln mix containing light and heavy Ln. For now, it remains unanswered how *P. alloputida* KT2440 recognizes and distinguishes between utilizable and non-utilizable Ln. In the future, coupling high-throughput microfluidic cultivation with downstream analyses, such as examining gene expression through RNAseq, might be a powerful strategy for studying Ln-related, phenotypic differences on multiple levels.

Observed growth patterns indicated that *P. alloputida* KT2440 interacts not only with light but also with heavy Ln. Intracellular Ln accumulation was previously shown for *M. extorquens* AM1 (18, 44) and Beijerinckiaceae bacterium RH AL1 (45). In the case of *M. extorquens* AM1, Ln are accumulated in the cytoplasm, whereas strain RH AL1 deposits Ln in the periplasm. In *M. extorquens* AM1 and Beijerinckiaceae bacterium RH AL1, Ln uptake into the peri- and cytoplasm is enabled by TonB-ABC transporter systems. The TonB-dependent receptor is in charge of uptake into the periplasm, and an ABC transporter facilitates translocation into the cytoplasm (18, 45, 46).

For *P. alloputida* KT2440, it was proposed that cytoplasmic Ln uptake relies on the PedA1A2BC (PP_2267–69, PP_5538) ABC transporter (22). We could neither identify extra- nor intracellular Ln accumulations in *P. alloputida* KT2440 through electron microscopy. Single-cell ICP-MS (47) enabled us to quantify cell-associated Ln. Cell-associated Lns in Ln-utilizing microbes have so far not been analyzed on the single-cell level. Despite only one known Ln-dependent protein, PedH, the determined values of cell-associated Ln (0.0025%–0.0068 % wet weight) are comparable to the total Fe content of a typical bacterial cell (48). We cannot distinguish between intra- and extracellular cell-associated Ln. Differences in the amount of cell-associated Ln are likely due to unspecific binding, governed by the intrinsic differences between Ln elements. Ln adsorption onto cell surfaces, with a preference for HREM, was previously shown for different organisms (49–51). We also showed this for Beijerinckiaceae bacterium RH AL1 (45). Unspecific extracellular binding, primarily through carboxyl and phosphoryl groups, skewed towards HREM, might facilitate the uptake of utilizable LREM.

Previous studies addressing Ln-utilizing methylotrophs revealed that Ln supplementation affected primarily *xox*- and *mxa*-cluster genes (52–55). *Methylobacterium aquaticum* 22A is the only microorganism featuring the Ln switch for which the effect of light (La) and heavy Ln (Ho, Lu) on gene expression was investigated over the whole transcriptome (53). Methylotrophy-related genes were sensitive to La addition, whereas Ho and Lu had no noticeable effect on gene expression. We could show that Ln have a tremendous effect on gene expression in Beijerinckiaceae bacterium RH AL1, which does not possess Ca-dependent PQQ ADH and lacks the Ln switch. Up to 41% of the encoded genes were differentially expressed in strain RH AL1 dependent on Ln supplementation (20).

We observed a clear difference between utilizable and non-utilizable Ln regarding gene expression. The upregulation of ribosomal protein genes L10 and L7/12; involved in ribosome stalk formation, translation factor recruitment, and GTP hydrolysis (56); when comparing La with Er and no Ln supplementation, suggested an overall effect on protein biosynthesis. The differential expression of numerous transcriptional regulator genes hinted towards dedicated mechanisms for transmitting changes in Ln availability. Genes encoding DUF2790-containing proteins were among the most differentially expressed genes dependent on Ln supplementation. This family of proteins of unknown function is conserved within the Pseudomonadaceae. The genome of *P. alloputida* KT2440 includes seven genes coding for DUF2790-containing proteins, of which four were responsive to Ln supplementation.

Some of the observed gene expression changes point towards Ln affecting the redox state of *P. alloputida* KT2440. Examples include the upregulation of genes linked to a spermidine/putrescine transport system when comparing La against Er and no Ln addition. Polyamines, such as spermidine and putrescine, function as antioxidants and signaling molecules (57, 58). When grown with La, Nd, or the Ln mix, genes associated with FeS cluster assembly were significantly more expressed, indicating higher demand, compared with cultures grown with Er or without Ln. Oxidative stress can cause an increased demand for functional FeS clusters (59). FeS cluster proteins are central to many forms of metabolism, including respiration, photosynthesis, as well as nitrogen fixation. FeS proteins can also function as sensors for intracellular oxygen and iron concentration (60).

Several genes for metal transporters showed differential gene expression when *P. alloputida* KT2440 was grown with Ln. Metalloproteins can accommodate different metals based on similar coordination geometry and the structure of the metal-binding site. Wehrmann and colleagues (22) could show that micromolar concentrations of Cu and Zn prevent the growth of a *P. alloputida* KT2440 *pedE* mutant when grown with nanomolar concentrations of Ln. Cu and Zn are known to strongly bind non-cognate metal-binding sites (61, 62).

*P. alloputida* KT2440 features two genes for phasin proteins (PP_5007, PP_5008), and one of them was significantly upregulated upon Ln supplementation. Scaffolds of phasin proteins surround polyhydroxybutyrate (PHB, a polyhydroxyalkanoate [PHA]) vacuoles (63). Besides from being linked to carbon storage, the presence of numerous proteins on their surface and their designation as carbonosomes highlights their assumed multifunctionality (64). PHB and related PHA are of economic relevance in the context of bioplastic production (65). For Beijerinckiaceae bacterium RH AL 1, we proposed that PHB plays a role in Ln uptake, periplasmic storage, and overall Ln homeostasis (20).

### Concluding remarks

We provide evidence that Ln sensing in *P. alloputida* KT2440 is tuned towards utilizable Ln. Our findings support the idea that the Ln switch operates as a fine-tuning switch, modulating the pool of Ca- and Ln-dependent PQQ dependent on Ln availability. Ln-dependent gene expression changes have been opaque but were arguably skewed towards metal homeostasis and changes in the overall cellular redox state. The observed differences in cell-associated Ln demand a deeper investigation to clarify how *P. alloputida* KT2440 interacts with and discriminates between Ln. The continued and in-depth study of Ln-utilization in organisms beyond methylotrophy is needed to fully unravel the relevance of Ln in microbial physiology and thus its potential impact on microbial ecology and potential biotechnological applications.

## MATERIALS AND METHODS

### Cultivation

*Pseudomonas alloputida* KT2440 (DSM #6125) was maintained on solid MP (*Methylobacterium* PIPES) medium (66) supplemented with 2-phenylethanol (5 mM) at 30°C. Ln used for cultivation were supplemented as trichloride salts (retrieved in analytical grade from Carl Roth [Karlsruhe, Germany] and Sigma-Aldrich [Taufkirchen, Germany]). Incubation experiments were carried out with MP medium either in 96-well plates (BRANDplates, Brand + CO. KG, Wertheim, Germany) (cultivation volume 300 µL) or acid-washed 150 mL Erlenmeyer flasks (cultivation volume 50 mL). Pre-cultures were grown with succinate as the carbon source (25 mM). More information is given in the supplemental material.

### Microfluidic cultivation

The setup used for microfluidic cultivation (Fig. S1) as well as its validation for two-dimensional screenings (Fig. S2) have been described in detail previously (67, 68). We used a syringe pump with six dosing units and a six-port droplet generator from Cetoni GmbH (Korbußen, Germany) to generate droplets with different compositions. Growth was tracked by recording the autofluorescence intensity *I*_*t*_, which was normalized according to the initial measurement intensity *I*_*t*0_. The background signal was determined based on the fluorinated-ethylene propylene (FEP) tubing filled with carrier liquid. Normalized autofluorescence units (NFU) were calculated as follows:


$${NFU}_{t}^{405nm/425nm}=\frac{I_{t}-I_{t0}}{I_{t0}}$$

After droplet formation, droplets were passed through the transparent FEP tubes with an outer diameter (OD) of 1.6 mm and an inner diameter (ID) of 0.5 mm for photofluorimetric analysis (*t* = 0) and subsequently collected in connected PTFE tube coils (ID 0.5 mm and OD 1.0 mm) for incubation. Additional details, including information about the validation of the setup, can be found in the supplemental material.

### Single-cell (sc) ICP-MS (inductively coupled plasma-mass spectrometry) analysis

Incubations were run as outlined before in 96-well plates, with three biological and six technical replicates. Incubations without Ln addition served as a control. Biomass was collected after the incubations reached OD_600_ values between 0.25 and 0.50. Technical replicates were pooled, harvested, and fixed with 500 µL of 2.5% (vol/vol) glutaraldehyde (in 100 mM cacodylate buffer) overnight at 4°C. Fixed and washed pellets were resuspended in 600 µL cacodylate buffer, and cell numbers were determined using a counting chamber. scICP-MS measurements were performed using an 8900 inductively coupled plasma-mass spectrometer (ICP-MS/MS) (Agilent Technologies, Inc., Santa Clara, CA, USA) equipped with a high-efficiency sample introduction system (Glass Expansion, Port Melbourne, Australia) composed of a total consumption spray chamber and a microconcentric nebulizer (47). Data acquisition is further outlined in the supplemental material. For determining the wet weight portion of Ln, we calculated with wet and dry weights of 1.7 g × L^−1^, and 0.4 g × L^−1^ per unit OD_600_ (69). Assuming a cell number of 7.8 × 10^8^ × (mL × OD_600_)^−1^ (70), gave us a wet weight per cell of approximately 2.2 pg.

### Electron microscopy

Transmission electron microscopy (TEM), and energy-dispersive X-ray spectroscopy (EDX) analyses were done as described previously (20, 45).

### RNA extraction, mRNA enrichment, and sequencing library preparation

RNA extraction and subsequent mRNA enrichment were done based on previously described methods (45, 46, 71). Sequencing libraries were prepared using the NEBNext Ultra II Directional RNA Library Prep Kit for Illumina (New England Biolabs, Germany). The size distribution of the libraries was checked by high-resolution gel electrophoresis with a Bioanalyzer instrument using the DNA 7500 Pico kit (Agilent Technologies). Libraries were quantified through fluorometry with a Qubit fluorometer and dsDNA HS reagents (Thermo Fisher Scientific, Darmstadt, Germany).

### Sequencing and data pre-processing

An equimolar pool of the prepared libraries was sequenced with a NovaSeq 6000 instrument (Illumina, San Diego, California, USA) in paired-end mode (2 × 100 bp). Sequencing was carried out by the sequencing core facility of the Leibniz Institute on Aging-Fritz Lipmann Institute (Jena, Germany). We assessed the quality of raw and trimmed sequences with FastQC (v0.11.9) (72). Adaptor- and quality-trimming (settings: minlen = 75, qtrim = rl, ktrim = rl, k = 25, mink = 11, trimq = 20) were carried out with bbduk (v38.26) (73) using its included database of common sequence contaminants and adapters. The filtering of rRNA-derived reads, the mapping of mRNA-derived reads, and the generation of the read count table for subsequent differential gene expression analysis are detailed in the supplemental material. The read count data were assessed based on pseudocount distributions, determined biological coefficients of variation, as well as *P*-value distributions obtained from testing for differential gene expression between replicate groups of RNAseq data sets (Fig. S7 to S9).

### Differential gene expression and cluster analysis

The R software framework for statistical computing (v4.2.1) and the package edgeR (v3.36.0) (74, 75) have been used to identify differentially expressed genes. We considered genes with changes in gene expression above |0.58| log_2_FC (FC), gene expression values higher than 4 log_2_counts per million (CPM), and FDR (false discovery rate) values smaller than 0.05 for differential gene expression analysis. Data exploration including cluster analysis is explained in more depth in the supplemental material.

### Quantitative reverse transcription-PCR (RT-qPCR)

RT-qPCR was used to assess gene expression changes regarding *pedE* and *pedH* independent from RNAseq-based analysis using the 2^-ΔΔ*CT*^ method (37). The 16S rRNA gene was used as a constitutively expressed reference gene. Biomass samples originating from repeated incubations, set up the same way as outlined above, were subjected to the previously described extraction procedure for recovering total RNA. Details regarding the reverse transcription, subsequent qPCR, used primers, and cycling conditions can be found in the supplemental material.

### Figure generation

We used the R software framework (v4.2.1) (76) for plotting and made use of the packages ggplot2 (v3.3.6) (77), gplots (v3.1.3) (78), ggpubr (v0.4.0) (79), cowplot (v1.1.1) (80), and upsetR (v1.4.0) (81), including their respective dependencies. Figures have been finalized with inkscape (https://inkscape.org/).

## Data Availability

RNAseq data sets are available via EBI/ENA ArrayExpress (accession: E-MTAB-13102) (https://www.ebi.ac.uk/arrayexpress/experiments/E-MTAB-13102). Details about data processing and analysis are also provided via the Open Science Framework (https://osf.io/ynsdc/). We also provide a reproducible, snakemake-based (82, 83) workflow for RNAseq data processing (https://github.com/wegnerce/smk_rnaseq, release v0.1).

## SUPPLEMENTAL MATERIAL

Supplemental Material msphere.00685-24-s0001.pdf Additional details regarding used methods, supplemental figures, and legends for supplemental tables.

Supplemental Tables msphere.00685-24-s0002.xls Tables S1 to S15.

ASM does not own the copyrights to Supplemental Material that may be linked to, or accessed through, an article. The authors have granted ASM a non-exclusive, world-wide license to publish the Supplemental Material files. Please contact the corresponding author directly for reuse.
