# Supplementary material for: Changes in growth, lanthanide binding, and gene expression in Pseudomonas alloputida KT2440 in response to light and heavy lanthanides
Source: mSphere. 2024 Sep 18;9(10):e00685-24. doi: 10.1128/msphere.00685-24 (PMC11520305; doi:10.1128/msphere.00685-24)
Supplement: Supplemental Material — Additional details regarding used methods, supplemental figures, and legends for supplemental tables. [file msphere.00685-24-s0001.pdf]

SUPPLEMENTARY MATERIAL: Changes in growth, lanthanide binding, and gene expression in *Pseudomonas alloputida* KT2440 in response to light and heavy lanthanides

Linda Gorniak[1], Sarah Luise Bucka[1], Bayan Nasr[2], Jialan Cao[2], Steffen Hellmann[3], Thorsten Schäfer[3], Martin Westermann[4], Julia Bechwar[1], Carl-Eric Wegner[1,5,#]

[1]Institute of Biodiversity, Aquatic Geomicrobiology, Friedrich Schiller University, Dornburger Str. 159, 07743 Jena, Germany

[2]Department of Physical Chemistry and Microreaction Technology, Institute for Chemistry and Biotechnique, Technische Universität Ilmenau, Weimarerstr. 32, 98693, Ilmenau, Germany

[3]Institute of Geosciences, Applied Geology, Friedrich Schiller University, Burgweg 11, 07749 Jena, Germany

[4]Electron Microscopy Center, Jena University Hospital, Ziegelmühlenweg 1, 07743 Jena, Germany

[5]current address: Department of Inorganic Chemistry, Bioinorganic Chemistry, Heinrich Heine University Düsseldorf, Universitätsstr. 1, 40225 Düsseldorf

[#]corresponding author: Carl-Eric Wegner | carl-eric.wegner@hhu.de

Running title: Lanthanide-induced growth and gene expression changes in *P. alloputida* KT2440

Keywords: lanthanides, lanthanome, RNAseq, microfluidics, single-cell ICP-MS

SUPPLEMENTARY MATERIAL

Supplementary Information. Complementary information about used materials and applied methods.

We also provide details about sequence data processing and differential gene expression analysis through an Open Science Framework (https://osf.io/) repository (https://osf.io/ynsdc/).

Cultivation

P. alloputida KT2440 was grown using MP (*Methylobacterium* PIPES) medium (1) with different La concentrations (10 nM, 50 nM, 100 nM, 250 nM, 500 nM, 1 µM, 2.5 µM, 5 µM, 10 µM) and 2-phenylethanol (5 mM) as the carbon source to determine the preferred La concentration range for growth. MP medium contained: 30 mM PIPES, 1.45 mM $K_2HPO_4 \times 3\ H_2O$, 1.88 mM $NaH_2PO_4 \times H_2O$, 0.5 mM $MgCl_2 \times 6\ H_2O$, 8 mM $(NH_4)_2SO_4$, 20 µM $CaCl_2 \times 2\ H_2O$, 45.6 µM sodium citrate, 1.2 µM $ZnSO_4 \times 7\ H_2O$, 1 µM $MnCl_2 \times 4\ H_2O$, 18 µM $FeSO_4 \times 7\ H_2O$, 2 µM $(NH_4)_6Mo_7O_{24} \times 4\ H_2O$, 1 µM $CuSO_4 \times 5\ H_2O$, 2 µM $CoCl_2 \times 6\ H_2O$, 0.33 $Na_2Wo_4 \times 2\ H_2O$. The pH of the medium was adjusted to 6.75. We subsequently tested growth with 50 nM of different Ln elements (La, Ce, Nd, Dy, Er, Ho, Yb, Mix [equimolar mix of Ce, Nd, Dy, Ho, Er, Yb; 50 nM total Ln], controls without added Ln). All incubations were monitored by spectrophotometry (λ = 600 nM). Monitoring of 96-well plates was done with a Synergy H4 Hybrid reader (Agilent Technologies, Waldbronn, Germany) (30°C, medium shaking) for 24 hours. For downstream RNAseq analysis, elemental analysis, and electron microscopy, incubations with selected Ln elements (50 nM

total Ln) were repeated in a volume of 50 mL in acid-washed Erlenmeyer flasks (150 mL) with cellulose stoppers. Biomass samples were collected during exponential growth, at optical densities between 0.17 and 0.33 (OD_{600nm}). Biomass samples were harvested by centrifugation (10 min, 10.000 × g, room temperature) and stored at -80°C until further processing.

Microfluidic cultivation

A highly-resolved, one-dimensional, dose-response screening was achieved by controlling the dosing of effectors, culture medium, and cell suspension into a carrier liquid stream (perfluoromethyldecalin, PP9). PTFE (polytetrafluoroethylene) tubing with an inner diameter of 0.5 mm and an outer diameter of 1.6 mm was used to connect the droplet generator to the computer-controlled syringe pump. We used syringes (ILS GmbH, Fürstenfeldbruck, Germany) with volumes of 500 µL for the effector solution (La, Er, Yb; 300 nM), 1000 µL for the cell suspension and culture medium (MP medium), and 10 mL for the PP9 carrier fluid. The generated droplets were transported through an optical detector unit at a constant flow rate of 200 µL/min, which facilitated measuring autofluorescence intensity data. Data acquisition was performed directly through the transparent PTFE tubing. Fluorescence measurements were performed using a laser diode with a peak wavelength of 405 nm (Changchun New Industries Optoelectronics, Changchun, PR China) and a long-pass filter (425 nm) (Laser Components, Olching, Germany) on the detection side. Emitted photons were collected using a photomultiplier module (Hamamatsu Photonics, Herrsching am Ammersee, Germany). Absorbance measurements were performed with four LEDs with peak wavelengths of 470, 505, 605, and 705 nm

(Agilent Technologies). A tube coil length of four meters with approximately 350 droplets was generated for the one-dimensional screening experiments. A tube coil length of six meters with approximately 600 droplets was generated for the two-dimensional screening experiments. The flow rates of the various fluids in the syringe pump were controlled using LAbVIEW (Natonal Instruments, USA). To manipulate the composition of droplets, the flow rates of the syringe pumps were adjusted according to the desired droplet fraction. In the case of investigating dose-response relationships for single substances, a stepwise increase in effector concentration with a syringe pump control program was employed to continuously change the desired effector concentration against a diluting medium. For two effectors (binary mixture), a stepwise increase in concentrations with a resolution of 10% was implemented. Consequently, for two effectors, 11 different concentration steps (0, 10, 20, 30, 40, 50, 60, 70, 80, 90, 100%) were combined, resulting in 121 different combinations. The flow rates of the carrier liquid and cell suspension were set at 136 µL/min and 32 µL/min, respectively. The flow rates of the effector solutions and cultivation medium varied within a total flow rate of 32 µL/min. Therefore, the overall flow rate of the segment generation process remained constant at 200 µL/min. An initial cell density of 2500 cells per 500 nL segment (10^7 cells × mL^{-1}) was used. The generation of the highly resolved one- and two-dimensional screening sequences took approximately four and nine minutes, respectively. After droplet formation, droplets were passed through the transparent FEP tubes with an outer diameter (OD) of 1.6mm and an inner diameter (ID) of 0.5 mm for photofluorimetric analysis (t = 0) and subsequently collected in connected PTFE tube coils (ID 0.5mm and OD 1.0 mm) for incubation. These tube coils were incubated for 3 days at room

temperature (21°C ± 2°C). The different droplet sequences were assessed after 2 and 3 days by passing through the photo-fluorimetric detection unit to monitor cell density and autofluorescence intensity. Details of the setup and data processing were described in our previous work (2, 3). The setup is illustrated in **Figure S1**. To analyze the precision achievable in a two-dimensional concentration space, we previously (4) validated the setup using effector model dyes. A flow program with stepwise increases in dye concentration was applied. To achieve a concentration resolution of 10%, eleven different concentration steps (0, 10, ... 100% of maximum effector concentration) were programmed, resulting in 11^2=121 combinations. Two anionic indicator dyes, cochineal red A (1 mM) and Orange G (1 mM), were used as aqueous solutions. The total flow rate of the aqueous phase (sum of the two effector solutions and the dilution medium) was set to 64 µL/min, and the flow rate of the carrier liquid was set to 136 µL/min. After droplet formation, they were transported at a constant flow rate of 200 µL/min through a microflow photometer equipped with two light-emitting diodes (LEDs) with peak wavelengths of 470 and 505 nm for monitoring the absorbance of the indicator dyes. The measurement beam had a diameter of approximately 0.5 mm, matching the internal diameter of the microtube. Integrating the optical signal over the entire residence time of the droplet ensured the registration of a representative optical signal for each cultivation volume. By applying the program shown in **Figure S2A**, we generated a sequence of about 800 droplets. The achieved normalized absorbance of both dyes is shown in **Figure S2B**. Each dot in **Figure S2B** represents the optical signal of one droplet. **Figure S2C** demonstrates that the effectively obtained two-dimensional dye distribution deviated marginally (±2.5%) from the expected values.

Single-cell (sc) ICP-MS (inductively coupled plasma-mass spectrometry) analysis

Fixed cell suspensions ($6.87 \times 10^7 - 5.15 \times 10^8$ cells × mL^{-1}) were diluted with ultrapure water to a final concentration of 10^4 cells × mL^{-1}. Samples were introduced using a Syringe Pump E-1000 (New Era Pump Systems Inc, Farmingdale, NY, USA) equipped with a 1 mL syringe (Hamilton, Bonaduz, Switzerland) with a constant flow rate of 10 µl × min^{-1}. All parameters are listed in **Table S14** and the ionic limits of detection (LOD) and quantification (LOQ) using 3, respectively, 10 × SD + mean (blank) criteria in **Table S15**. A total acquisition time of 2 min and a dwell time of 5 ms in time-resolved analysis mode were used. All chosen isotopes ($^{31}P^+$, $^{139}La^+$, $^{140}Ce^+$, $^{144}Nd^+$ and $^{166}Er^+$) were measured in separate runs using oxygen gas in the reaction cell with a mass shift to $^{31}P^{16}O^+$, $^{139}La^{16}O^+$, $^{140}Ce^{16}O^+$, $^{144}Nd^{16}O^+$ and $^{166}Er^{16}O^+$ in triple quadrupole mode to prevent ICP-MS based interferences. P was used for tracking bacterial cells, as it is highly abundant in cell constituents such as phospholipids. Au nanoparticles (NPs, as $^{197}Au^+$ in No gas mode) with a nominal diameter of 30 nm (LGCQC5050, LGC, London, UK) at a final concentration of 2 × 10^4 NPs × mL^{-1} were used to determine the transport efficiency from the syringe to the plasma via particle number method described previously (5). The ionic calibrations were prepared for the quantification in 1 mM EDTA (pH 8) to stabilize the ions in a range from 0 to 10 µg × L^{-1}.

Sequence data pre-processing

Trimmed reads were filtered with SortMeRNA (v2.1) (6) and the SILVA (7) and Rfam (8) databases by removing rRNA-derived and non-coding RNA sequences. Read

mapping onto the reference genome of *P. alloputida* KT2440 was done with *bbmap* (v.38.26) (settings: slow, k = 11). The number of mapped reads per feature (e.g. coding genes) was determined from *.bam* files, that have been sorted and indexed with *samtools* (v1.3.1) (9), using *featureCounts,* which is part of the *Subread* package (v1.6.3) (10).

Differential gene expression analysis

Pseudo-counts (log_2(counts+1)) (**Figure S7**) were generated for exploring read count data using MA (mean of the normalized counts versus the log_2FC [fold change] for all genes tested) plots. Multidimensional scaling plots were generated with *limma* (v3.50.0) (11). Scaled read count data was used to examine inter-dataset relationships. Scatterplot matrices were plotted with the *plotSM* function of *bigPint* (v. 1.10.0) (12, 13) to check correlations between intra- and inter-sample replicates. Read counts were filtered to remove genes, which recruited insufficient numbers of reads. Filtering was done with the *filterByExpr* function that is part of *edgeR* (v3.36.0) (14, 15). Samples were normalized based on calculated normalization factors and assessed dispersion. We estimated the uncertainty of transcript abundances based on replicates by calculating the biological coefficient of variation (BCV) (**Figure S8**). We determined p-values deduced from the exact test established by Robinson and Smyth (16) for all carried-out comparisons (**Figure S9**).

Cluster analysis

Based on the observed differences in gene expression, we were interested to which extent genes were co-expressed/-regulated. We used K-means clustering (17) for

grouping genes in clusters based on similar gene expression patterns across the different incubation conditions. K-means clustering represents a form of partitional clustering, meaning that genes are grouped into K partitions. This requires *a priori* knowledge about the number of clusters. Higher numbers of K lead to smaller cluster sizes, making data exploration more granular, while smaller K values tend to oversimplify the data. We used different algorithms to define a suitable value for K. Used algorithms included the sum of squared error, average silhouette width, the Calinski-Harabasz index, the gap statistic, and hierarchical clustering (18–22) (**Figure S4**).

Quantitative reverse-transcription PCR (RT-qPCR)

Extracted total RNA from an independent incubation was reverse-transcribed using the GoScript^{Tm} Reverse Transcription System (Promega, Mannheim, Germany), following the manufacturer's protocol, and using random hexamers as primers. PCR primers for *pedE* (*pedE_F* 5'-TTG TGT TCC CAC ACC AGC TT-3', *pedE_R* 5'-TGT TCT ACA TCC CCG GCA AC-3') and *pedH* (*pedH_F* 5'-ATG CCG ATG GCC TAC AAC AA-3', *pedH_R* 5'-ATT GAG CGG CTT GAT GGT GA-3') were designed using *NCBI Primer-Blast* (https://www.ncbi.nlm.nih.gov/tools/primer-blast/) starting from the respective gene sequences (*pedE*, locus tag: PP_RS13925; *pedH*, locus tag: PP_RS13950; NCBI RefSeq *P. alloputida* KT2440 genome accession: NC_002947.4). For quantifying 16S rRNA gene transcripts, we used a previously published (23, 24) primer pair (*Bac8Fmod* 5'-AGA GTT TGA TYM TGG TCTC AG-3'; *Bac338Rabc* 5'-GCW GCC WCC CGT AGG WGT-3'). Transcripts were quantified in triplicates with Brilliant II SYBR® Green qPCR Master Mix (Agilent, Waldbronn,

Germany) using a Bio-Rad CFX Real-Time PCR Detection System (Bio-Rad, Feldkirchen, Germany). The cycling conditions were 95°C for 10 min, followed by 40 cycles of 30s at 95°C, 30 s at 59.3°C (*pedE*) or 56.4°C (*pedH*), and 60 s at 72°C. For the assay targeting the 16S rRNA gene, the cycling conditions have been: 95°C 10 min, followed by 40 cycles of 30 s at 95°C, 1 min at 55°C, 25s at 72°C, and 10s at 78°C. Fluorescence signals were recorded after the annealing and extension steps. Standard curves were constructed using plasmids with cloned *pedE* and *pedH* fragments from *P. alloputida* KT2440, or cloned 16S rRNA gene fragments from freshwater environments. All standard curves were linear from 5×10^8 to 50 copies for all three target genes, with R^2 above 0.99 and PCR efficiencies between 90 and 100%. PCR specificity was assessed by melt curve analysis. Observed threshold cycles of no-enzyme controls of the reverse-transcription reaction showed that the background caused by undigested DNA was negligible. Changes in gene expression were determined by applying the $2^{-\Delta\Delta CT}$ method (25).

Figure S1. Experimental droplet-based microfluidic setup for 1D and 2D-screening of cultivation parameters. On the left side of the illustration, there is a 6-channel syringe pump and a droplet generator responsible for generating the droplets. The droplets, consisting of aqueous cell media, are separated from each other by an immiscible continuous phase. The size/volume, spacing, and composition of the droplets can be adjusted by controlling the flow rate program of the syringe pump. A combined photo-fluorimetric micro flow-through detector unit is used to measure the generated droplets. Subsequently, the droplet sequences are collected and incubated in PTFE tube coils.

Figure S2. Analysis of the droplet composition accuracy for two-dimensional screenings (in 11 concentration steps resulting in 121 concentration points) with stepwise varied compositions of two effector model dyes (cochineal red A [Col. red A] and Orange G). A syringe pump program for the generation of two-dimensional concentration spaces in 11 concentration steps resulting in 121 concentration combinations was applied. Droplet absorbances were measured for the two dyes (B). Droplet concentration composition accuracy based on measured dye concentration combinations are shown as normalized absorbance/absorbance plot (C). The shown data were previously reported (4) and the shown figure is based on Figure 9 in the referenced publication (4).

Figure S3. Transmission electron microscopy and energy-dispersive X-ray spectroscopy (EDX) of *P. alloputida* KT2440. *P. alloputida* KT2440 was grown in MP medium (1) with 5 mM 2-phenylethanol as the carbon source and supplemented with

either La, Nd, or without Ln. We did two rounds of incubations, one with 50 nM and one with 1 µM of the respective Ln. Cells were inspected for intracellular biominerals as indicator for local Ln accumulation (A, scale bar = 500 nm). Control cultures without added Ln were monitored and analyzed for both rounds of incubations. We only subjected samples from 1 µM incubations to EDX-based elemental analyses (B). We did not consider single cells, but defined a bigger measuring field, which enabled the analyses of whole groups of cells. The upper part of (B) shows inverted micrographs, green circles indicate the defined measuring field. The lower half shows deconvoluted EDX spectra. Ln could not be detected. (k)eV = (kilo) electron volt, cps = counts per second.

Figure S4. Growth curves of *P. alloputida* KT2440 incubations for downstream RNAseq-based gene expression analysis. Samples for qPCR analysis were collected at time points t_0-t_3. Biomass for RNA extraction was collected at t_1 (La, Nd, Ln mix) and t_2 (Er, none [= no Ln controls]). Line and dot shapes refer to the different elements. Incubations were done in triplicates (n = 3 biological replicates). Plotted are average OD_{600nm} values and respective standard deviations.

Figure S5. Algorithms used to define a suitable K for clustering differentially expressed genes by *K-means* clustering. Tested algorithms included hierarchical clustering based on *Spearman* distances (A), the Calinski-Harabasz index (B) (21), sum of squared errors (C), average silhouette width (ASW) (D) (20), and the gap statistic (E) (22). K=6 is marked in all subpanels. WSS = weighted sum of squares.

Figure S6. Relative expression of the target genes *pedE* and *pedH* in response to light (La) and heavy Ln (Er), determined by reverse transcription qPCR. The upper panel shows the $2^{-\Delta\Delta Ct}$ values of *pedE* and *pedH*, which were calculated by using the 16S rRNA gene for normalization and samples without Ln supplementation as controls.

Figure S7. Comparison of the different RNAseq data sets based on pseudocount distributions. Pseudocounts were determined gene-wise by calculating Log_2([count of mapped reads]+1).

Figure S8. Determined biological coefficients of variation (BCV). The BCV gives an estimate for the coefficient of variation with which the true abundance of transcripts varies within replicate groups of RNAseq data sets. Log_2CPM = log_2 counts per million.

Figure S9. P-value distributions obtained from testing for differential gene expression between replicate groups of RNAseq data sets. The corresponding p-values have been deduced from the exact test proposed by Robinson and Smyth (16), which is implemented in *edgeR (14)*.

Table S1. Growth characteristics determined by means of *growthcurver*. K = carrying capacity, n0 = initial population size at the beginning of the growth curve, r = intrinsic growth rate (h-1), t_gen = generation time (h), t_gen_min = generation time (min),

auc_l = area under the logistic curve, auc_e = empirical area under the curve, sigma = goodness of fit.

Table S2. Autofluorescence data from the microfluidic two-dimensional screening La vs. Er. 3-6 microfluidic segments have been incubated per combination of La and Er. Autofluorescence values represent averages for all segments of a given combination. SD = standard deviation, % SD = standard deviation as a percentage of the average values.

Table S3: Overview of gene expression data of *Pseudomonas alloputida KT2440* grown with phenylethanol (5 mM) as carbon source, and supplemented with either 50 nM of La, Nd, Er, a Ln mix (Ce, Nd, Dy, Ho, Er, Yb; equimolar pooled). Gene expression is given in log_2 counts per million (CPM). Given annotation data were retrieved from NCBI RefSeq and KEGG GENES. La.1-3 = KT2440 grown with 50 nM La, Nd.1-3 = KT2440 grown with 50 nM Nd, Er.1-3 = KT2440 grown with 50 nM Er, Mix.1-3 = KT2440 grown with an equimolarly pooled cocktail of heavy and light lanthanides (50 nM).

Table S4: Overview of differentially expressed genes in *P. alloputida* KT2440 when grown with either 50 nM La or without added Ln. Given annotation data were retrieved from NCBI RefSeq and KEGG GENES. logFC = log_2 Foldchange, logCPM = log_2 Counts per million, FDR = false discovery rate.

Table S5: Overview of differentially expressed genes in *P. alloputida* KT2440 when grown with either 50 nM Nd or 50 nM La. Given annotation data were retrieved from NCBI RefSeq and KEGG GENES. logFC = log_2 Foldchange, logCPM = log_2 Counts per million, FDR = false discovery rate.

Table S6: Overview of differentially expressed genes in *P. alloputida* KT2440 when grown with either 50 nM Er or 50 nM La. Given annotation data were retrieved from NCBI RefSeq and KEGG GENES. logFC = log_2 Foldchange, logCPM = log_2 Counts per million, FDR = false discovery rate.

Table S7: Overview of differentially expressed genes in *P. alloputida* KT2440 when grown with either 50 nM Ln mix or 50 nM La. Given annotation data were retrieved from NCBI RefSeq and KEGG GENES. logFC = log_2 Foldchange, logCPM = log_2 Counts per million, FDR = false discovery rate.

Table S8: Overview of differentially expressed genes in *P. alloputida* KT2440 when grown with either 50 nM Nd or without added Ln. Given annotation data were retrieved from NCBI RefSeq and KEGG GENES. logFC = log_2 Foldchange, logCPM = log_2 Counts per million, FDR = false discovery rate.

Table S9: Overview of differentially expressed genes in *P. alloputida* KT2440 when grown with either 50 nM Er or 50 nM Nd. Given annotation data were retrieved from NCBI RefSeq and KEGG GENES. logFC = log_2 Foldchange, logCPM = log_2 Counts per million, FDR = false discovery rate.

Table S10: Overview of differentially expressed genes in *P. alloputida* KT2440 when grown with either 50 nM Ln mix or 50 nM Nd. Given annotation data were retrieved from NCBI RefSeq and KEGG GENES. logFC = $\log_2$ Foldchange, logCPM = $\log_2$ Counts per million, FDR = false discovery rate.

Table S11: Overview of differentially expressed genes in *P. alloputida* KT2440 when grown with either 50 nM Er or without added Ln. Given annotation data were retrieved from NCBI RefSeq and KEGG GENES. logFC = $\log_2$ Foldchange, logCPM = $\log_2$ Counts per million, FDR = false discovery rate.

Table S12: Overview of differentially expressed genes in *P. alloputida* KT2440 when grown with either 50 nM Ln mix or 50 nM Er. Given annotation data were retrieved from NCBI RefSeq and KEGG GENES. logFC = $\log_2$ Foldchange, logCPM = $\log_2$ Counts per million, FDR = false discovery rate.

Table S13: Overview of differentially expressed genes in *P. alloputida* KT2440 when grown with either 50 nM Ln mix or without added Ln. Given annotation data were retrieved from NCBI RefSeq and KEGG GENES. logFC = $\log_2$ Foldchange, logCPM = $\log_2$ Counts per million, FDR = false discovery rate.

Table S14: ICP-MS/MS 8900 operating conditions for single-cell mode.

Table S15: Estimation of ionic limit of detection (LOD) and quantification (LOQ) for scICP-MS. For the estimation, > ten blank signals and the slope of the calibration curve were used to calculate the LODs/LOQs (LOD = 3 × standard deviation + mean; LOQ = 10 × standard deviation + mean.

__**Note:**__ All supplementary tables are provided in one combined spreadsheet.

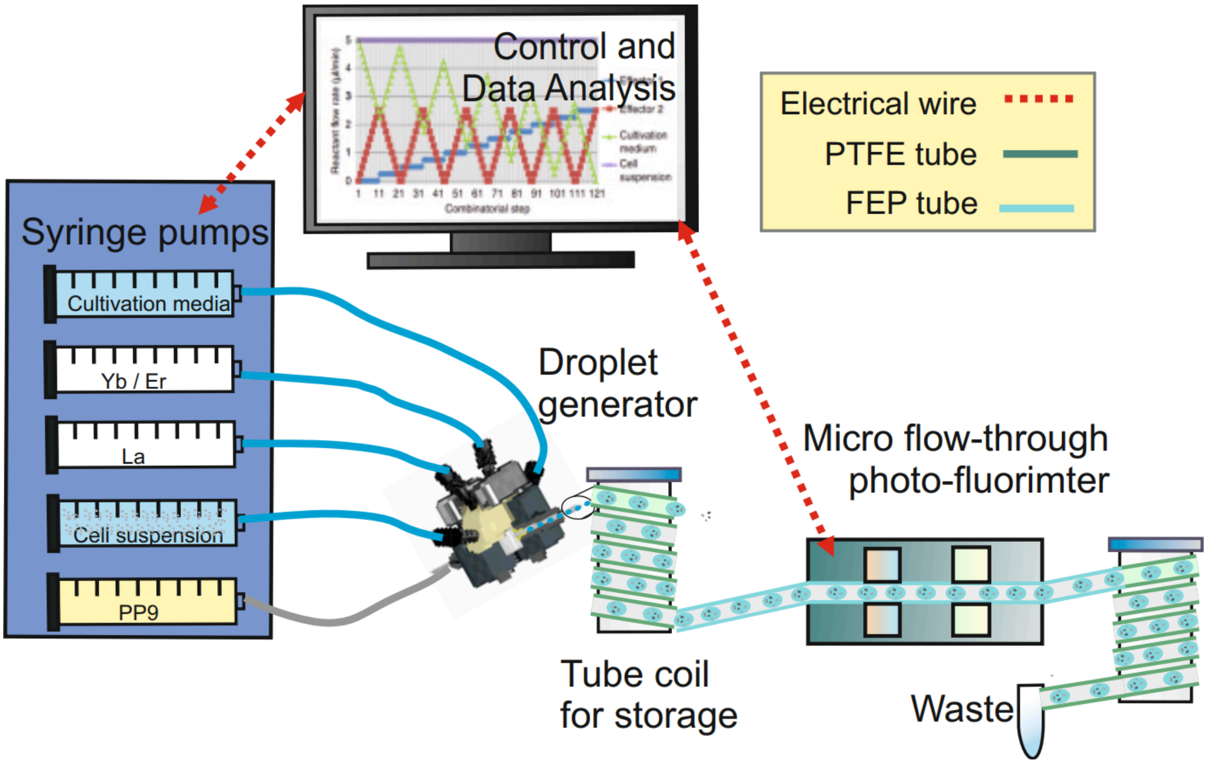

Control and
Data Analysis
Electrical wire
PTFE tube
FEP tube
Syringe pumps
Cultivation media
Yb / Er
La
Cell suspension
PP9
Droplet
generator
Micro flow-through
photo-fluorimter
Tube coil
for storage
Waste

Figure S1.

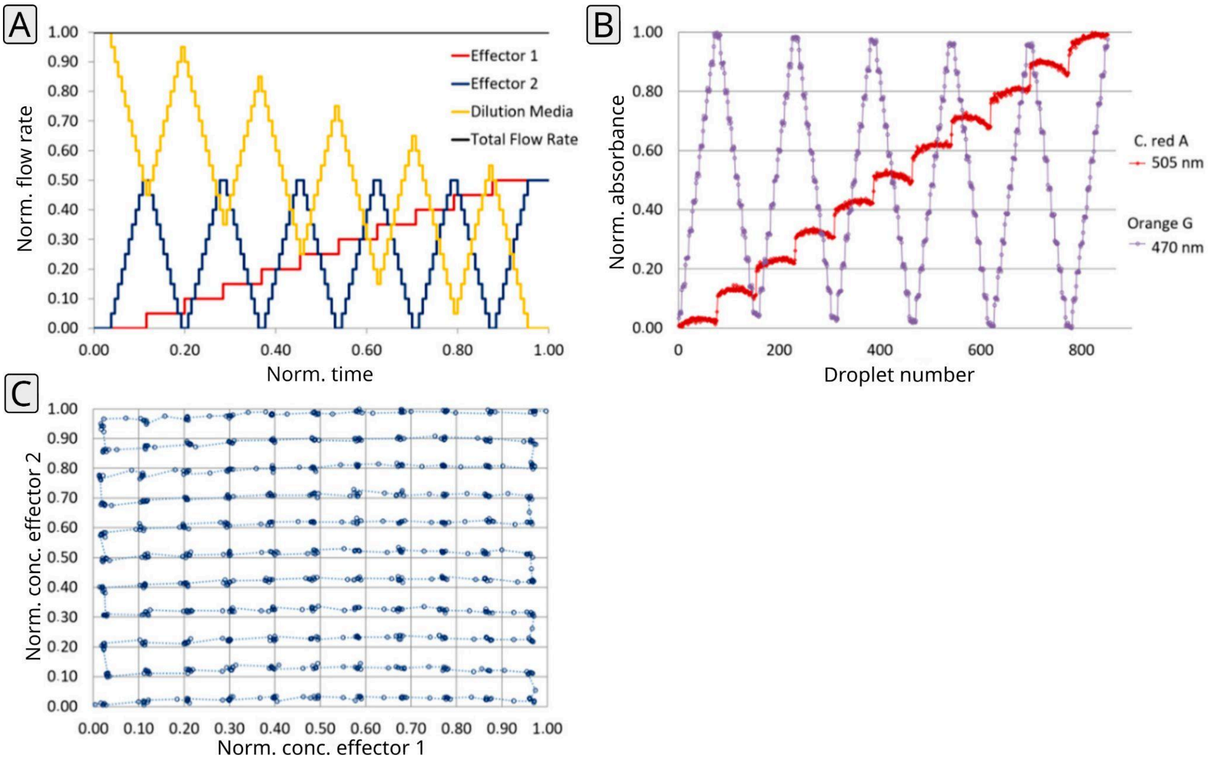

A
B
C
Effector 1
Effector 2
Dilution Media
Total Flow Rate
Norm. flow rate
Norm. time
C. red A
505 nm
Orange G
470 nm
Norm. absorbance
Droplet number
Norm. conc. effector 2
Norm. conc. effector 1

Figure S2.

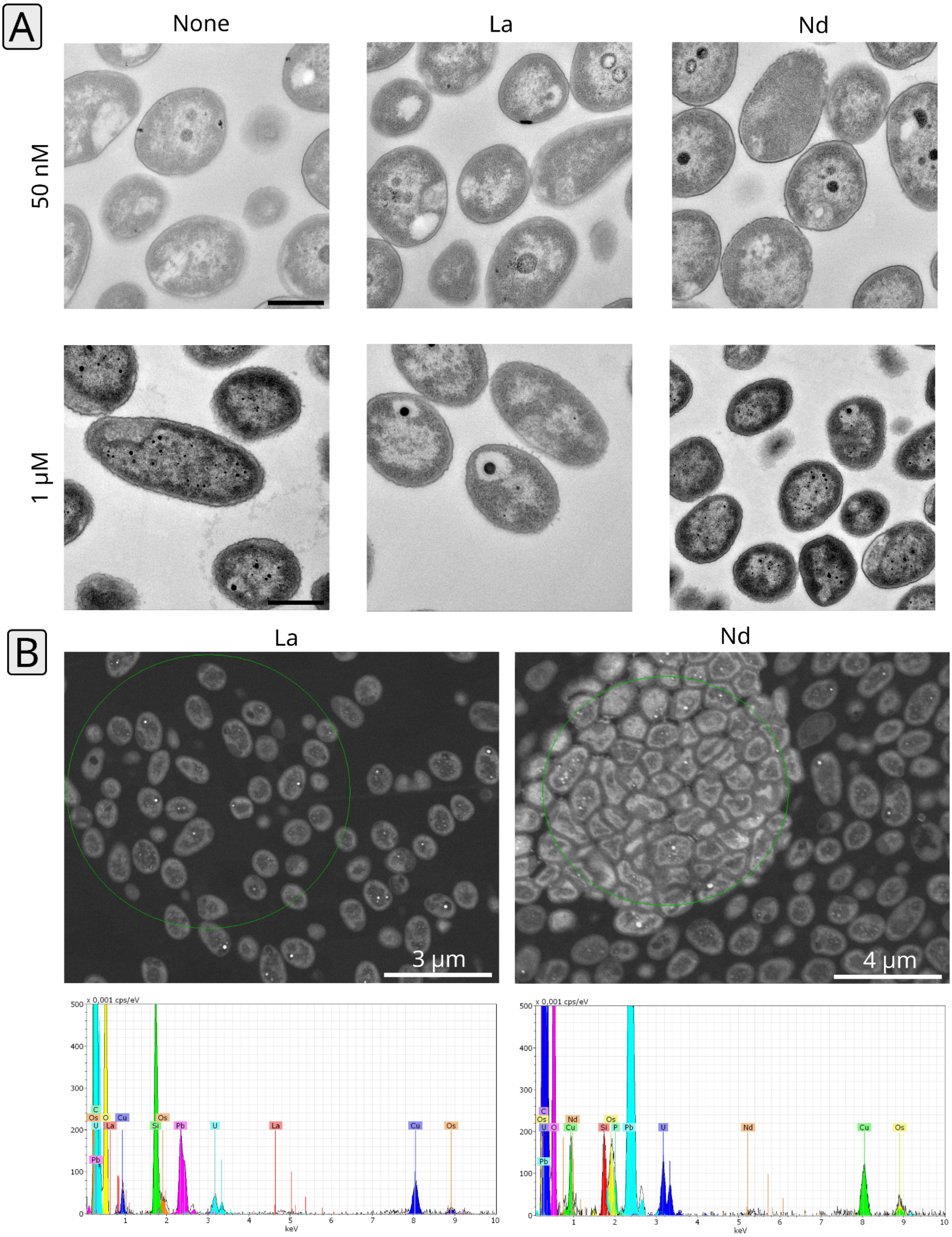
A
None
La
Nd
50 nM
1 µM
B
La
Nd
3 µm
4 µm
x 0,001 cps/eV
keV
x 0,001 cps/eV
keV

S3

Figure S3.

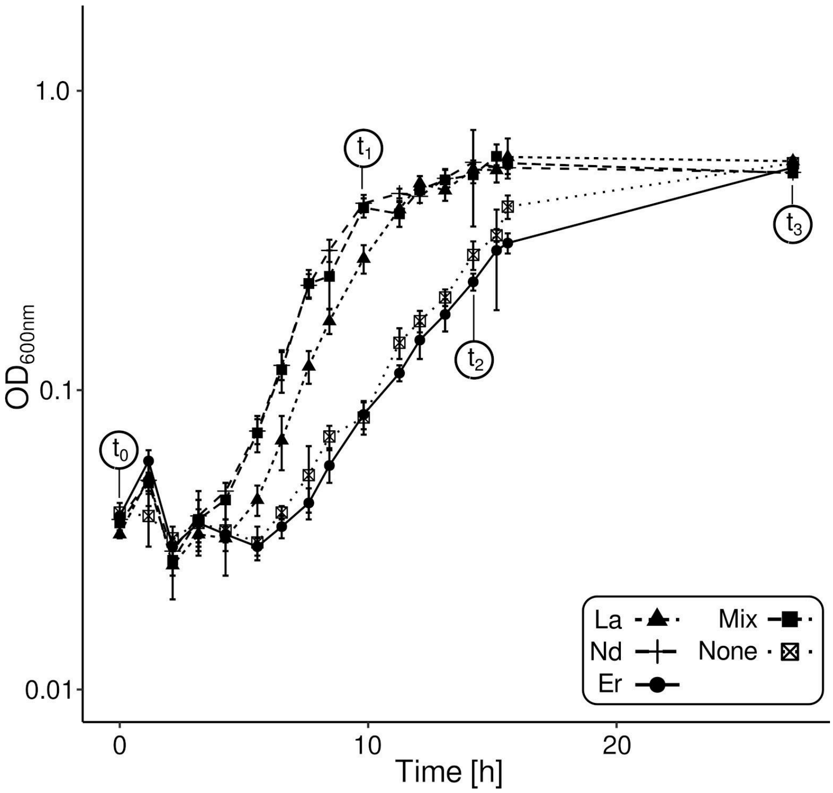

Figure S4.

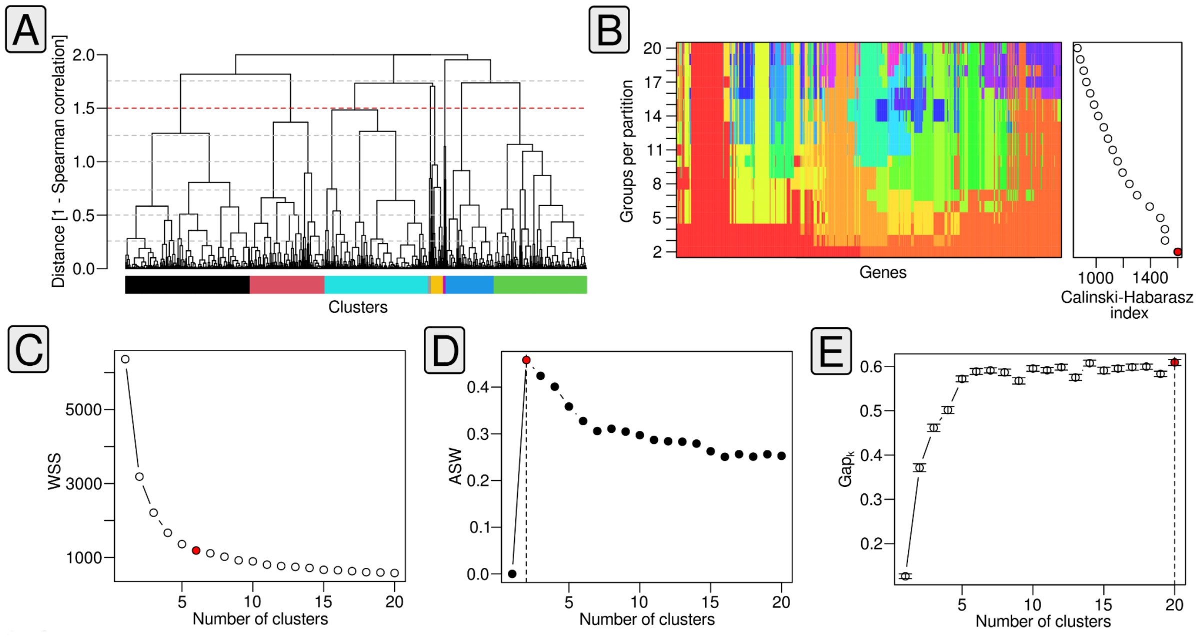
A
B
C
D
E
Distance [1 - Spearman correlation]
Clusters
Groups per partition
Genes
Calinski-Habarasz index
WSS
ASW
Gap
Number of clusters

Figure S5.

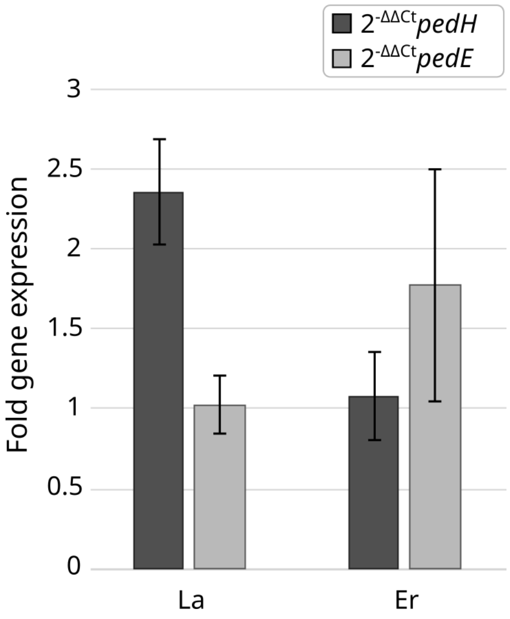
2-ΔΔCt pedH
2-ΔΔCt pedE
3
2.5
2
1.5
1
0.5
0
Fold gene expression
La
Er

Figure S6.

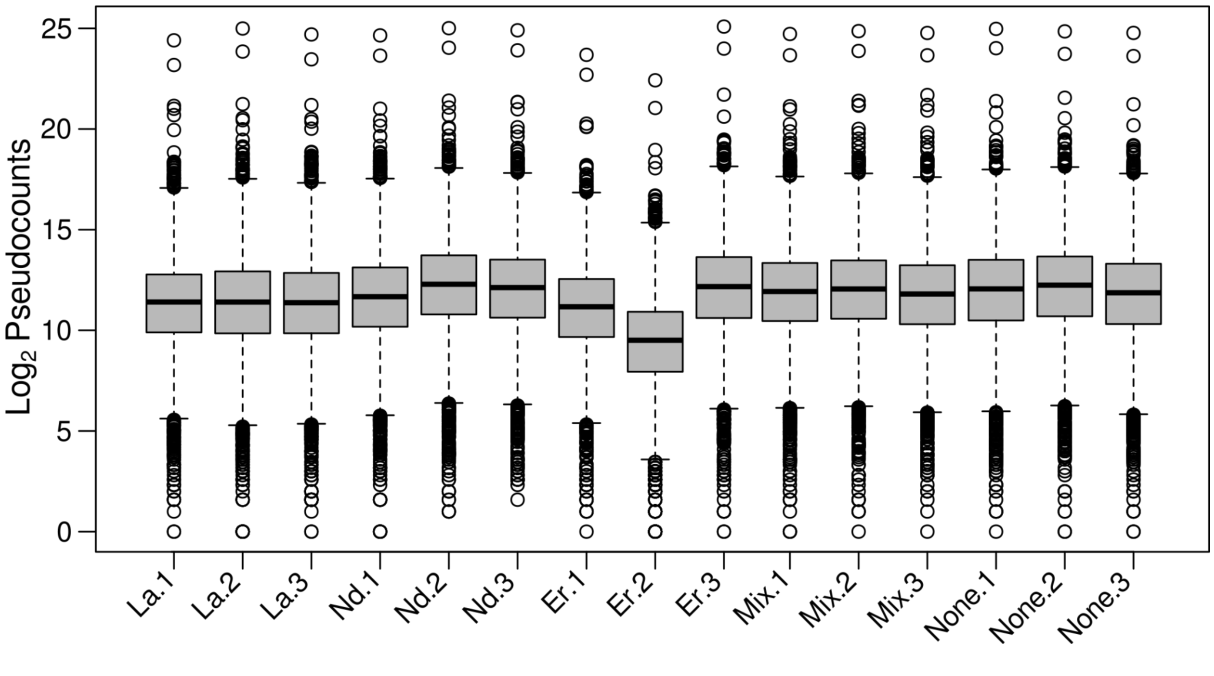
Log2 Pseudocounts
0
5
10
15
20
25
La.1
La.2
La.3
Nd.1
Nd.2
Nd.3
Er.1
Er.2
Er.3
Mix.1
Mix.2
Mix.3
None.1
None.2
None.3

Figure S7.

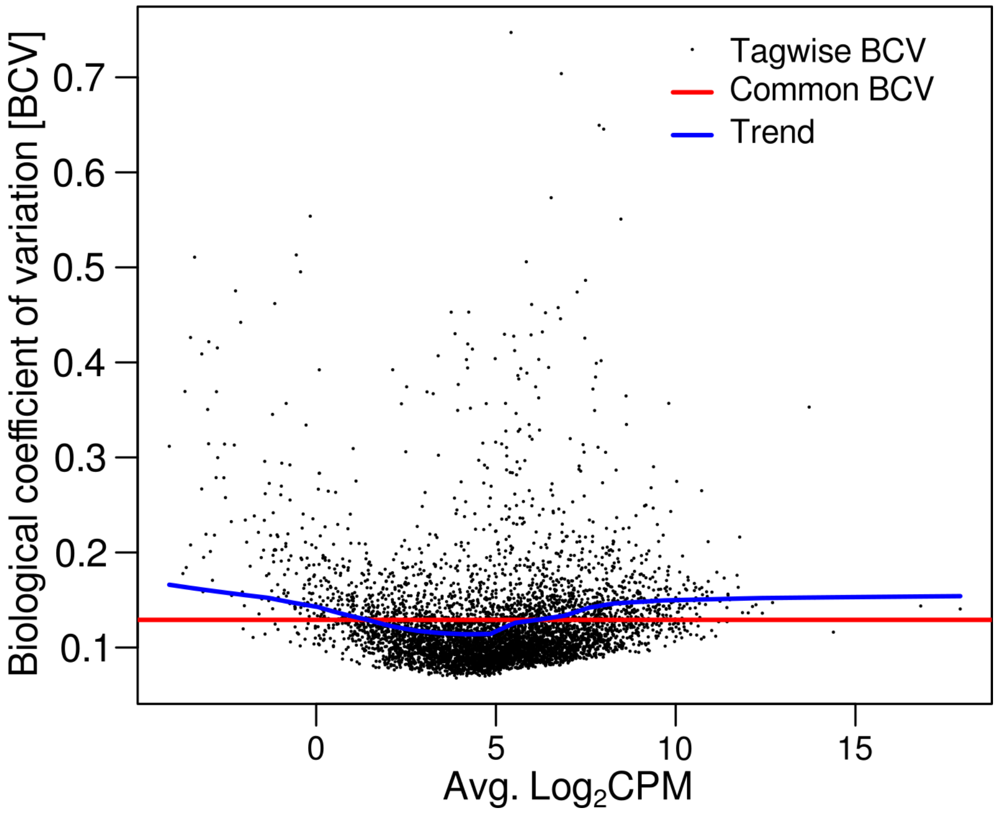
Tagwise BCV
Common BCV
Trend
Biological coefficient of variation [BCV]
0.1
0.2
0.3
0.4
0.5
0.6
0.7
0
5
10
15
Avg. Log_2CPM

Figure S8.

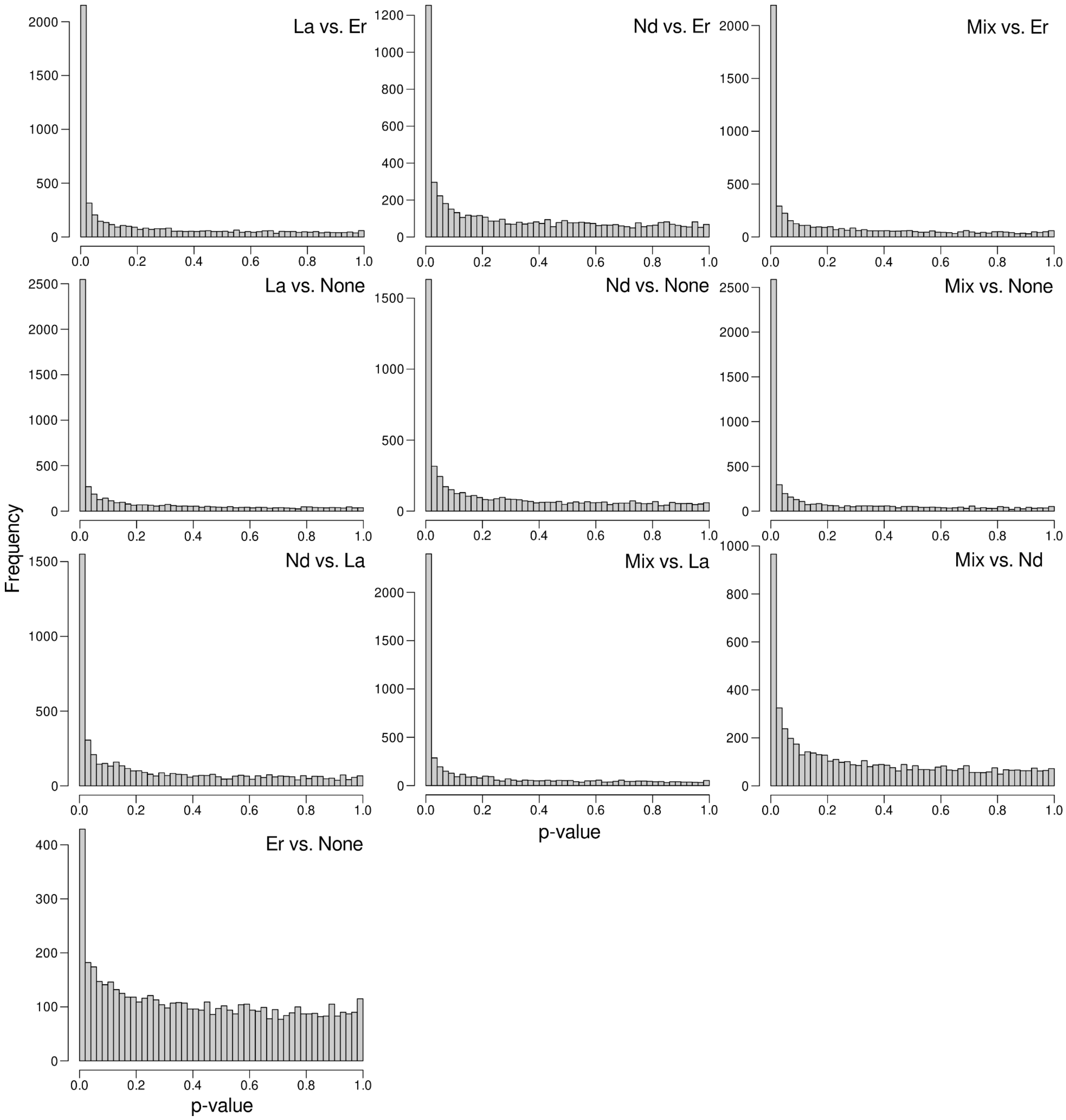
La vs. Er
Nd vs. Er
Mix vs. Er
La vs. None
Nd vs. None
Mix vs. None
Nd vs. La
Mix vs. La
Mix vs. Nd
Er vs. None
Frequency
p-value

Figure S9.

References

1. Delaney NF, Kaczmarek ME, Ward LM, Swanson PK, Lee M-C, Marx CJ. 2013. Development of an optimized medium, strain and high-throughput culturing methods for Methylobacterium extorquens. PLoS One 8:e62957.
2. Cao J, Kürsten D, Schneider S, Knauer A, Günther PM, Köhler JM. 2012. Uncovering toxicological complexity by multi-dimensional screenings in microsegmented flow: modulation of antibiotic interference by nanoparticles. Lab Chip 12:474–484.
3. Cao J, Russo DA, Xie T, Groß GA, Zedler JAZ. 2022. A droplet-based microfluidic platform enables high-throughput combinatorial optimization of cyanobacterial cultivation. Sci Rep 12:15536.
4. Cao J, Richter F, Kastl M, Erdmann J, Burgold C, Dittrich D, Schneider S, Köhler JM, Groß GA. 2020. Droplet-based screening for the investigation of microbial nonlinear dose-response characteristics system, background and examples. Micromachines 11:577.
5. Montaño MD, Olesik JW, Barber AG, Challis K, Ranville JF. 2016. Single Particle ICP-MS: Advances toward routine analysis of nanomaterials. Anal Bioanal Chem 408:5053–5074.
6. Kopylova E, Noé L, Touzet H, Noe L, Touzet H. 2012. SortMeRNA: fast and accurate filtering of ribosomal RNAs in metatranscriptomic data. Bioinformatics 28:3211–3217.
7. Yilmaz P, Parfrey LW, Yarza P, Gerken J, Pruesse E, Quast C, Schweer T, Peplies J, Ludwig W, Glöckner FO. 2014. The SILVA and "All-species Living Tree Project (LTP)" taxonomic frameworks. Nucleic Acids Res 42:D643–8.
8. Burge SW, Daub J, Eberhardt R, Tate J, Barquist L, Nawrocki EP, Eddy SR, Gardner PP, Bateman A. 2013. Rfam 11.0: 10 years of RNA families. Nucleic Acids Res 41:D226–32.
9. Li H, Handsaker B, Wysoker A, Fennell T, Ruan J, Homer N, Marth G, Abecasis G, Durbin R, 1000 Genome Project Data Processing Subgroup. 2009. The Sequence Alignment/Map format and SAMtools. Bioinformatics 25:2078–2079.
10. Liao Y, Smyth GK, Shi W. 2013. The Subread aligner: fast, accurate and scalable read mapping by seed-and-vote. Nucleic Acids Res 41:e108.
11. Ritchie ME, Phipson B, Wu D, Hu Y, Law CW, Shi W, Smyth GK. 2015. limma powers differential expression analyses for RNA-sequencing and microarray studies. Nucleic Acids Res 43:e47.
12. Rutter L, Moran Lauter AN, Graham MA, Cook D. 2019. Visualization methods for differential expression analysis. BMC Bioinformatics 20:458.
13. Rutter L, Cook D. 2020. bigPint: A Bioconductor visualization package that makes big data pint-sized. PLoS Comput Biol 16:e1007912.
14. Robinson MD, McCarthy DJ, Smyth GK. 2010. edgeR: a Bioconductor package for differential expression analysis of digital gene expression data. Bioinformatics 26:139–140.
15. McCarthy DJ, Chen Y, Smyth GK. 2012. Differential expression analysis of multifactor RNA-Seq experiments with respect to biological variation. Nucleic Acids Res 40:4288–4297.
16. Robinson MD, Smyth GK. 2008. Small-sample estimation of negative binomial dispersion, with applications to SAGE data. Biostatistics 9:321–332.
17. Jin X, Han J. 2010. K-Means Clustering, p. 563–564. *In* Sammut, C, Webb, GI (eds.), Encyclopedia of Machine Learning. Springer US, Boston, MA.
18. Defays D. 1977. An efficient algorithm for a complete link method. Comput J 20:364–366.
19. James G, Witten D, Hastie T, Tibshirani R. 2021. An introduction to statistical learning: With applications in R, 2nd ed. Springer, New York, NY.
20. Rousseeuw PJ. 1987. Silhouettes: A graphical aid to the interpretation and validation of cluster analysis. J Comput Appl Math 20:53–65.
21. Caliński T, Harabasz J. 1974. A dendrite method for cluster analysis. Commun Stat Simul Comput 3:1–27.
22. Tibshirani R, Walther G, Hastie T. 2001. Estimating the Number of Clusters in a Data Set Via the Gap Statistic. J R Stat Soc Series B Stat Methodol 63:411–423.
23. Daims H, Brühl A, Amann R, Schleifer KH, Wagner M. 1999. The domain-specific probe EUB338 is insufficient for the detection of all Bacteria: development and evaluation of a more comprehensive probe set. Syst Appl Microbiol 22:434–444.
24. Loy A, Lehner A, Lee N, Adamczyk J, Meier H, Ernst J, Schleifer K-H, Wagner M. 2002. Oligonucleotide microarray for 16S rRNA gene-based detection of all recognized lineages of sulfate-reducing prokaryotes in the environment. Appl Environ Microbiol 68:5064–5081.

25. Livak KJ, Schmittgen TD. 2001. Analysis of relative gene expression data using real-time quantitative PCR and the 2(-Delta Delta C(T)) Method. Methods 25:402–408.
